# CD47 Agonist Peptides Induce Programmed Cell Death in Refractory Chronic Lymphocytic Leukemia B Cells via PLCγ1 Activation: Evidence from Mice and Humans

**DOI:** 10.1371/journal.pmed.1001796

**Published:** 2015-03-03

**Authors:** Ana-Carolina Martinez-Torres, Claire Quiney, Tarik Attout, Heloïse Boullet, Linda Herbi, Laura Vela, Sandrine Barbier, Danielle Chateau, Elise Chapiro, Florence Nguyen-Khac, Frédéric Davi, Magali Le Garff-Tavernier, Roba Moumné, Marika Sarfati, Philippe Karoyan, Hélène Merle-Béral, Pierre Launay, Santos A. Susin

**Affiliations:** 1 Cell Death and Drug Resistance in Lymphoproliferative Disorders Team, INSERM UMRS1138, Centre de Recherche des Cordeliers, Paris, France; 2 Sorbonne Universités, Université Pierre et Marie Curie Paris 6, UMRS1138, Centre de Recherche des Cordeliers, Paris, France; 3 Université Paris Descartes, Sorbonne Paris Cité, UMRS1138, Centre de Recherche des Cordeliers, Paris, France; 4 INSERM U1149, Paris, France; 5 Faculté de Médecine, Site Xavier Bichat, Université Paris Diderot, Sorbonne Paris Cité, Paris, France; 6 Laboratoire des Biomolécules, UMR 7203 and FR 2769, Sorbonne Universités, Université Pierre et Marie Curie, Paris, France; 7 Centre National de la Recherche Scientifique, UMR 7203, Paris, France; 8 Département de Chimie, École Normale Supérieure, Paris, France; 9 Intestine: Nutrition, Barrier, and Diseases Team, INSERM U1138, Centre de Recherche des Cordeliers, Paris, France; 10 Service d’Hématologie Biologique, Groupe Hospitalier Pitié-Salpêtrière, Assistance Publique—Hôpitaux de Paris, Paris, France; 11 Immunoregulation Laboratory, Centre de Recherche du Centre Hospitalier de l’Université de Montréal, Montréal, Quebec, Canada; Kyoto University, JAPAN

## Abstract

**Background:**

Chronic lymphocytic leukemia (CLL), the most common adulthood leukemia, is characterized by the accumulation of abnormal CD5^+^ B lymphocytes, which results in a progressive failure of the immune system. Despite intense research efforts, drug resistance remains a major cause of treatment failure in CLL, particularly in patients with dysfunctional *TP53*. The objective of our work was to identify potential approaches that might overcome CLL drug refractoriness by examining the pro-apoptotic potential of targeting the cell surface receptor CD47 with serum-stable agonist peptides.

**Methods and Findings:**

In peripheral blood samples collected from 80 patients with CLL with positive and adverse prognostic features, we performed *in vitro* genetic and molecular analyses that demonstrate that the targeting of CD47 with peptides derived from the C-terminal domain of thrombospondin-1 efficiently kills the malignant CLL B cells, including those from high-risk individuals with a dysfunctional *TP53* gene, while sparing the normal T and B lymphocytes from the CLL patients. Further studies reveal that the differential response of normal B lymphocytes, collected from 20 healthy donors, and leukemic B cells to CD47 peptide targeting results from the sustained activation in CLL B cells of phospholipase C gamma-1 (PLCγ1), a protein that is significantly over-expressed in CLL. Once phosphorylated at tyrosine 783, PLCγ1 enables a Ca2^+^-mediated, caspase-independent programmed cell death (PCD) pathway that is not down-modulated by the lymphocyte microenvironment. Accordingly, down-regulation of PLCγ1 or pharmacological inhibition of PLCγ1 phosphorylation abolishes CD47-mediated killing. Additionally, in a CLL-xenograft model developed in NOD/scid gamma mice, we demonstrate that the injection of CD47 agonist peptides reduces tumor burden without inducing anemia or toxicity in blood, liver, or kidney. The limitations of our study are mainly linked to the affinity of the peptides targeting CD47, which might be improved to reach the standard requirements in drug development, and the lack of a CLL animal model that fully mimics the human disease.

**Conclusions:**

Our work provides substantial progress in (i) the development of serum-stable CD47 agonist peptides that are highly effective at inducing PCD in CLL, (ii) the understanding of the molecular events regulating a novel PCD pathway that overcomes CLL apoptotic avoidance, (iii) the identification of PLCγ1 as an over-expressed protein in CLL B cells, and (iv) the description of a novel peptide-based strategy against CLL.

## Abstract 2

In this study, Santos Susin and colleagues demonstrate that a serum-stable CD47 agonist peptide is highly effective at inducing apoptosis in chronic lymphocytic leukemia B-cells and mice.

## Editors’ Summary

**Background:**

Chronic lymphocytic leukemia (CLL) is the most common type of adult leukemia (cancer of the white blood cells). It accounts for about a third of all adult leukemias, and about one in 200 people will develop CLL during their lifetime, usually in old age. White blood cells (including T and B lymphocytes) are made by the bone marrow (the soft center of certain bones in the body) and help the body fight infections. Leukemia begins when a bone marrow cell acquires genetic changes (mutations and chromosomal abnormalities) that make it grow and develop abnormally. Over time, the abnormal cells accumulate in the bone marrow, blood, and lymphoid organs (lymph nodes and spleen) (malignant B lymphocytes accumulate in the case of CLL) and stop the marrow producing healthy blood cells. CLL does not usually cause any symptoms during its early stages and is often diagnosed as a result of a routine blood test. Some patients, however, develop painless swellings in the lymph nodes in the neck, armpit, or groin. Other symptoms of CLL include tiredness, frequent infections, and weight loss. Treatments for CLL, which are not given unless symptoms develop, include chemotherapy, immunotherapy, and targeted therapies, which attack cancer cells without harming normal cells.

**Why Was This Study Done?:**

CLL develops very slowly, and people with CLL usually live for many years after their diagnosis. However, there is no cure for CLL, and treatment fails in up to a quarter of patients because resistance to chemotherapy develops. The outlook (prognosis) is particularly bad for patients whose cancer cells contain a dysfunctional *TP53* gene (*TP53* encodes a protein that normally regulates cell division and cell death). Although some new therapies have recently been approved in the US and Europe, additional approaches to the treatment of CLL are still needed. One potential target for new treatments is the cell surface receptor CD47. In CLL cells, activation of CD47 by a small fragment (peptide) of thrombospondin-1 (a protein that binds to CD47) called 4N1K rapidly induces caspase-independent programmed cell death (PCD), a type of cell death in which the cell uses specialized cellular machinery to kill itself. (PCD normally controls cell numbers and eliminates cells that threaten an animal’s survival, but cancer cells often resist PCD.) Unfortunately, peptides are usually degraded quickly in the human body, which limits their usefulness as therapeutic agents. Here, therefore, the researchers investigate whether PKHB1, a variant of 4N1K designed to be stable in the body (a “serum-stable” agonist peptide), might provide a new approach to the treatment of CLL.

**What Did the Researchers Do and Find?:**

The researchers tested the ability of PKHB1 to kill malignant CLL B lymphocytes grown from blood samples collected from 80 patients with CLL. PKHB1 efficiently killed these cells, including those from individuals with a dysfunctional *TP53* gene but, importantly, did not kill either normal T and B lymphocytes collected from healthy donors or the residual normal T and B lymphocytes present in the patient samples. Genetic and molecular analyses undertaken by the researchers indicated that the differential response of normal and leukemic B lymphocytes to PKHB1 was the result of sustained activation of a protein called phospholipase C gamma-1 (PLCγ1) in the malignant CLL B lymphocytes. Other experiments showed that PLCγ1 activation drove the induction of a new PCD pathway (a calcium-mediated, caspase-independent pathway) that was not down-regulated by survival stimuli provided by the lymphocyte microenvironment. Finally, the researchers report that injection of PKHB1 reduced the tumor burden in a mouse model of CLL.

**What Do These Findings Mean?:**

Taken together, these findings suggest that serum-stable CD47 agonist peptides represent a potential new treatment for CLL that could circumvent the problem of chemotherapy resistance and eliminate malignant CLL B lymphocytes while sparing residual normal B and T lymphocytes. These findings also demonstrate that PKHB1, the serum-stable CD47 agonist peptide tested in this study, directly induces a novel PCD pathway, thereby overcoming the innate avoidance of apoptosis by CLL cells. Before peptide-based strategies for the treatment of CLL can enter clinical trials, however, further studies are needed to develop peptides with increased stability and increased ability to bind to and activate CD47. Moreover, because the mouse CLL model used here incompletely recreates the development of CLL in people, the effectiveness and safety of this pioneer approach needs to be tested much more rigorously in animals before any clinical trials are initiated.

**Additional Information:**

Please access these websites via the online version of this summary at http://dx.doi.org/10.1371/journal.pmed.1001796.

- The US National Cancer Institute provides information about cancer and how it develops, about targeted cancer therapies, and about CLL and its treatment (in English and Spanish)
- Cancer Research UK, a not-for-profit organization, provides general information about cancer and how it develops and detailed information about CLL
- The UK National Health Service website also provides information about CLL
- The American Society for Hematology provides general information about blood cancer and detailed information about leukemia, including CLL
- The French Society for Hematology provides general information about CLL (in French)
- The not-for-profit Force Hemato organization provides general information about lymphoproliferative disorders and resources for patients and families (in French)
- The not-for-profit organization HealthTalkOnline provides personal stories about living with CLL
- Wikipedia provides information about programmed cell death and about CD47 (note that Wikipedia is a free online encyclopedia that anyone can edit; available in several languages)

## Introduction

Chronic lymphocytic leukemia (CLL), a human malignancy caused by an imbalance between proliferation and programmed cell death (PCD) [1], is the most common form of leukemia in adults. CLL is characterized by an accumulation of monoclonal B cells (CD20^+^, CD5^+^, and CD23^+^) in the peripheral blood, bone marrow, and secondary lymphoid organs that leads to the progressive failure of the immune and hematopoietic systems [2]. CLL prognosis is dependent on clinical Rai (United States) or Binet (Europe) staging and biological markers, including *IGHV* status, cytogenetic abnormalities, *NOTCH1* or *SF3B1* mutations, and the expression of proteins such as CD38 or ZAP70 [3–5]. Despite intense research and pharmaceutical development, CLL remains an incurable disease. Indeed, 15%–25% of patients remain or become refractory to the current chemotherapeutic regimes [6]. Moreover, patients with a dysfunctional *TP53* gene (~7%) require a specific aggressive therapy that often yields negative results [7]. Novel therapies targeting the B cell receptor (BCR)–associated kinases have recently been approved in the United States and Europe for relapsed CLL [8,9]. However, it is still very important to develop alternative PCD approaches that kill specifically the malignant CLL cells, including those in high-risk individuals, while sparing the residual CD5^−^ B lymphocytes and the T cells of the CLL patient.

From this perspective, CD47 appears to be a target of high therapeutic potential in CLL. CD47, a cell surface receptor that binds to signal regulatory protein-α (SIRPα) and thrombospondin-1 (TSP1), serves as a marker of self and participates in the regulation of the cellular responses to stress [10–17]. The binding of CD47 to either SIRPα or TSP1 provides two anticancer strategies. On the one hand, antibodies targeting CD47 or SIRPα, recombinant SIRPα, or TSP1-derived proteins promote phagocytosis and tumor cell elimination by disrupting the CD47–SIRPα interaction and/or inducing Fc-dependent mechanisms or PCD [15,17–28]. On the other hand, the binding of CD47 to 4N1K, a decapeptide derived from the TSP1 C-terminal domain [10,16,29–31], kills tumor cells and inhibits tumor growth *in vivo* [23,32–37]. Even if peptide-based strategies have so far been underused, a renewed interest in therapeutic peptides has emerged because of advances in the development of novel tools aimed at generating peptides that are resistant to proteolytic degradations (e.g., obtained by introduction of amino acid surrogates or non-natural amino acids) [38]. Of 340 potential therapeutic peptides described to date, 160 (60 in phase I, 80 in phase II, and 20 in phase III) are being clinically investigated. In addition, there are more than 80 approved peptides (58 are drugs and 23 are used as diagnostic agents or vaccines). Note that the overall success rate of peptide drugs from phase I to commercial launch is approximately 25%, which is comparable to the success rate of biologic drugs and twice as high as that of small molecules (~10%–12%) [38]. Moreover, peptides have fewer side effects than biologics and are more cost-effective. Indeed, peptides such as goserelin, octreotide, and lanreotide are commonly used anticancer drugs [38]. Altogether, the success in the development of efficient therapeutic peptides emphasizes the medical and pharmaceutical relevance of a peptide-based approach against CLL [6,7].

In view of the above, here we test the cytotoxic effect of serum-stable CD47 peptide agonists in primary CLL B cells. Then, we use a complementary molecular and cell biology approach to unravel the mechanisms regulating the PCD pathway enabled by these peptides in the malignant B lymphocytes.

## Methods

### Patients, B Cell Purification, and Culture Conditions

The procedures in our study were in accordance with the Helsinki Declaration and were approved by the ethical committee on human experimentation at Pitié-Salpêtrière Hospital (CPPIDF6, Paris, France). After obtaining written consent, peripheral blood was collected from 80 patients diagnosed with CLL according to classical morphological and immunophenotypic criteria. These criteria include clinical Binet staging and the biological parameters *IGHV* mutational status and CD38 and ZAP70 levels. Deletions of 17p13, 11q22 and 13q14 and trisomy 12 were detected by fluorescence in situ hybridization (FISH) using the Vysis LSI p53/LSI ATM and LSI D13S319/LSI 13q34/CEP 12 Multi-color Probe Kits (Abbott Molecular). The functional status of *TP53* was determined by flow cytometry, as previously described [39]. *TP53* mutations were detected using high resolution melting (HRM). The amplicons with an abnormal HRM profile were confirmed by sequencing.

Mononuclear cells were purified from blood samples using a standard Ficoll-Hypaque gradient, and B cells were positively or negatively selected by magnetic microbeads coupled either to an anti-CD19 monoclonal antibody (positive selection) or to anti-CD16,-CD3, and-CD14 monoclonal antibodies (negative depletion) (Miltenyi Biotech). No differences in cell death response were encountered in positively or negatively selected cells. B lymphocytes, MEC-1, and M210B4 bone marrow stromal cells (ATCC) were cultured in complete medium (RPMI 1640 supplemented with 10% fetal calf serum, 2 mM L-glutamine, and 100 U/ml penicillin-streptomycin). In selected experiments, CLL cells were cultured in Ca^2+^-free medium (RPMI 1640 without calcium, supplemented with dialyzed 10% fetal calf serum) prior to the induction of cell death.

### Peptide Synthesis

Peptides were synthesized by solid-phase peptide synthesis on preloaded Fmoc-Lys(Boc)-Wang or Fmoc-D-Lys(Boc)-Wang resins (Merck Chemicals). The syntheses were performed on an ABI 433A peptide synthesizer (Applied Biosystems) at the 0.25-mmol scale. Peptides were purified by reverse phase high-performance liquid chromatography (HPLC) on an ACE (C8, 5 μm, 300 Å, 10 mm × 250 mm) column (Waters) with a gradient elution (0.1% [v/v] TFA in acetonitrile) in aqueous 0.1% (v/v) TFA. Homogeneous fractions were pooled and lyophilized after confirming purity greater than 95% by analytical HPLC.

**PKHB1, 4N1K, and 4NGG**. All Nα-Fmoc amino acids (10 equiv.) were coupled after activation with HBTU (0.45 M in NMP) in the presence of DIEA (6 equiv., 2 M in NMP; 10 equiv., 1:1). N-Fmoc deprotection was performed using piperidine (20% in NMP) and was monitored by measuring the UV absorbance of the released N-(9-fluorenylmethyl)-piperidine group at 301 nm. PKHB1, MALDI-TOF MS: k-R-F-Y-V-V-M-W-K-k MH+ calculated: 1,383.8; MH+ actual: 1,385.3. 4N1K, MALDI-TOF MS: K-R-F-Y-V-V-M-W-K-K MH+ calculated: 1,383.8; MH+ actual: 1,385.0. 4NGG, MALDI-TOF MS: K-R-F-Y-G-G-M-W-K-K MH+ calculated: 1,300.7; MH+ actual: 1,300.4.

### Peptide Degradation Assays

4N1K or PKHB1 (10 mg/ml), diluted in a 1:4 human serum/RPMI 1640 mixture, was incubated at 37°C for different lengths of time, then mixed with ethanol and 5 ml of 1 M NaOH and incubated at 4°C for at least 15 min to precipitate serum proteins. The supernatant was collected and injected in an HPLC system, and the soluble peptide was eluted by a linear gradient of 5% to 50% ACN (0.1% [v/v] TFA in acetonitrile) in aqueous 0.1% (v/v) TFA. The concentration of the peptide was calculated by the integration of the absorbance at 220 nm as a function of the retention time.

### Flow Cytometry

Annexin-V-APC (0.1 μg/ml; BD Biosciences) was used for the assessment of phosphatidylserine exposure, propidium iodide (PI, 0.5 μg/ml) for cell viability analysis, and tetramethylrhodamine ethyl ester (TMRE, 20 nM) for mitochondrial transmembrane potential (ΔΨ_m_) quantification. PCD was recorded in a FACSCanto II (BD Biosciences) in the total population (10,000 cells), and data were analyzed using FlowJo software. Chymotrypsin-like serine protease (serpase) or caspase cytofluorometric detection was performed with SerPase or Caspase activity kits from Imgenex (IMI-2301 and IMI-2315). Residual and leukemic B cells from CLL patients were discriminated from the mononuclear blood fraction by double labeling with anti-CD19 PerCP-Cyc5.5 (clone SJ25C1; BD Biosciences) and anti-CD5-PE-Cy7 mAb (clone L17F12; BD Biosciences). T cells from CLL patients were identified by a double anti-CD19 PerCP-Cyc5.5 and anti-CD3-PE-Cy7 (clone SK7; BD Biosciences) staining. Calreticulin cell surface exposure was recorded with anti-calreticulin-PE (clone FMC75; Assay Designs). CD47 analysis was assessed with conjugated anti-CD47-PE (clone B6H12; BD Biosciences), P21 with anti-P21-FITC (clone EA10; Calbiochem), and P53 with anti-P53-PE (clone DO-7; BD Biosciences). A QuantiBRITE flow cytometry system (BD Biosciences) was used to assess the number of CD47 molecules expressed on normal and CLL B lymphocytes.

### Cell Death Induction and Inhibition

We mainly used fresh CLL samples presenting low levels of spontaneous apoptosis. To induce PCD, 1 × 10^6^ cells/ml were treated for 2 h with PKHB1 (200 μM), 4N1K (300 μM), 4NGG (300 μM), or an anti-CD47 monoclonal antibody (5 mg/ml, clone B6H12 in soluble or immobilized conditions). To provide pro-survival microenvironment signals, CLL cells were pre-incubated with sCD40L (5 μg/ml) and IL-4 (20 ng/ml) or co-cultured with the M210B4 bone marrow stromal cell line (ATCC) before death was induced. To control for caspase-dependent apoptosis, cells were incubated for 12 h with etoposide (250 μM). For the inhibition assays, BAPTA-AM (20 μM); BAPTA (5 mM); Ru360 (750 nM); dantrolene (40 μM); 2-aminoethoxydiphenyl borate (2-APB, 60 μM); U73122 (200 nM); the broad spectrum caspase inhibitors Q-VD-OPh (QVD, 10 μM) and z-VAD-FMK (50 μM); the specific caspase inhibitors z-DEVD-FMK (50 μM, caspase-3/7), z-LEHD-FMK (50 μM, caspase-8), and z-IETD-FMK (50 μM, caspase-9); the serpase inhibitor TPCK (20 μM); or the fusion protein hSIRPα-Fc (15 μg/ml) [40,41] was added 30 min before inducing PCD.

### Protein Extraction and Immunoblotting

Cell fractions were lysed in 20 mM Tris-HCl (pH 7.4), 150 mM NaCl, 1% Triton X-100, and 1 mM EDTA supplemented with anti-protease and anti-phosphatase cocktails (Roche). Mitochondrial fraction was obtained with the help of a kit from Pierce. The protein concentration was determined using the Bio-Rad DC kit, and 70 μg of protein was loaded in linear SDS-PAGE gels. After blotting, nitrocellulose filters were probed with primary antibodies against CD47 (clone 2D3; eBioscience), activated caspase-3 (9661; Cell Signaling Technology), PLCγ1 (clone D9H10; Cell Signaling Technology), PLCγ1-Y783 (2821; Cell Signaling Technology), Cox IV (clone 1D6E1A8; Life Technologies), DRP1/DLP1 (clone 8/DLP1; BD Biosciences), and α-tubulin (clone B-5–1–2; Sigma). Immunoreactive proteins were detected using HRP-conjugated secondary antibodies and visualized with the ECL system (Thermo Scientific). Immunoblot images were acquired on a MF-ChemiBIS 4.2 (DNR Bio-Imaging Systems). PLCγ1-Y783 was quantified using Multi Gauge 3.0 software (Fujifilm Life Sciences). The optical density was normalized to the background and was expressed relative to the untreated cells (set at 1.0).

### Electron Microscopy

CLL cells were centrifuged for 10 min at 300*g*. The pellets obtained were fixed at 4°C for 2 h in 2.5% glutaraldehyde in 0.1 M phosphate buffer (pH 7.30), postfixed for 1 h in 1% buffered osmium tetroxide, dehydrated through a graded ethanol series, and embedded in Epon 812. Ultrathin sections were counterstained with 2% aqueous uranyl acetate for 30 min, then with lead citrate for 10 min, and viewed under a Philips 100× electron microscope.

### Ca^2+^ Measurement

Intracellular Ca^2+^ mobilization was assessed as previously described [42,43]. B cells were loaded for 30 min at 37°C with 1 μM Fura2-AM and pluronic acid (both from Life Technologies) in glass bottom dishes (MatTek Corporation) and washed in Ringer’s solution (145 mM NaCl, 5.4 mM KCl, 2 mM CaCl2, 1 mM MgCl2, 10 mM glucose, 10 mM HEPES, and 0.1% BSA [pH 7.5] with NaOH) or, in particular cases, with 5 mM BAPTA. Then, the B cells were treated with PKHB1 or ionomycin (maximum response) at the indicated concentrations and were excited by wavelengths of 340 and 380 nm. The fluorescence emissions of several cells were simultaneously recorded at a frequency of 1 Hz using a dual excitation fluorometric imaging system (TILL Photonics) and TILLvisION software. Signals were computed into relative ratio units of the florescence intensity of the different wavelengths (340/380 nm). Fluorescence values were analyzed using Origin software (OriginLab) and were normalized to the first value according to the equation (*F*/*F*_0_) − 1, where *F* is the fluorescence at a specific time point and *F*_0_ is the fluorescence at time 0. The area under the curve, representing the extent of Ca^2+^ mobilization, was calculated for each cell and graphed.

### Fibrillar and Globular Actin Assessment

The fibrillar/globular actin ratio was determined by fluorometric assessment. Excitation/emission filters were 485/538 and 544/590 nm for Phalloidin-FITC (F-actin) and DNase-Alexa 594 (G-actin), respectively. One unit equals the basal F-actin/G-actin ratio measured in 10^6^ untreated cells. All reactions were recorded in an Infinite M1000 PRO plate reader (Tecan).

### Quantitative Real-Time PCR

Total RNA was extracted from 11 normal and 50 CLL B lymphocyte samples using the Nucleospin RNA II kit (Macherey-Nagel). cDNA was prepared using Superscript II reverse transcriptase (Life Technologies). Quantitative real-time PCR (RT-PCR) was performed using TaqMan Gene Expression Assays (Life Technologies) for *CD47*, *PLCB1 (phospholipase C*, *beta 1*), *PLCB2* (*phospholipase C*, *beta 2*), *PLCB3 (phospholipase C*, *beta 3*), *PLCB4 (phospholipase C*, *beta 4*), *PLCG1 (phospholipase C*, *gamma 1*), *PLCG2 (phospholipase C*, *gamma 2*), *ITPR1 (inositol 1*,*4*,*5-trisphosphate receptor*, *type 1*), *ITPR2 (inositol 1*,*4*,*5-trisphosphate receptor*, *type 2*), *ITPR3 (inositol 1*,*4*,*5-trisphosphate receptor*, *type 3*), *RYR1 (ryanodine receptor 1*), *RYR2 (ryanodine receptor 2)*, *RYR3 (ryanodine receptor 3)*, *STIM1 (stromal interaction molecule 1)*, *STIM2 (stromal interaction molecule 2)*, *ORAI1 (calcium release-activated calcium modulator 1)*, *ORAI2 (calcium release-activated calcium modulator 2)*, and *ORAI3 (calcium release-activated calcium modulator 3)*. PCR reactions were performed in triplicate using TaqMan Universal PCR Master Mix (Life Technologies). The products were amplified in a ViiA7 Real-Time PCR System (Life Technologies) at 60°C for 40 cycles. Data were analyzed using the comparative threshold cycle method. The expression of *GUSB* or *ABL* [44] was utilized to normalize the data.

### PLCγ1-Y783 Flow Cytometry Assessment

At various time points after PKHB1 treatment, 5 × 10^5^ cells were fixed in ethanol and permeabilized in Triton X-100. Then cells were saturated in PBS + Triton X-100 + 10% FCS, incubated with anti-PLCγ1-Y783 (Cell Signaling Technology), and detected using an anti-rabbit IgG conjugated with Alexa Fluor 488. The data on the entire cell population were collected on a FACSCanto II flow cytometer. PLCγ1-Y783 was quantified based on the mean fluorescence intensity (MFI) for each sample.

### IP_3_ Quantification

IP_3_ was measured using IP_1_ as a surrogate [45] with an HTRF assay (Cisbio). The assays were performed in triplicate in 96-well plates, and the signal was quantified on an Infinite M1000 PRO plate reader (Tecan).

### Vectors and Lentiviral Transduction

To down-regulate PLCγ1, we utilized two short hairpin RNAs (shRNAs) (A: CTGAGAAATACGTGAACAA; B: AGTGAATGCTAGACAGAAA) and a control scrambled shRNA (ACGATAGTCGGTCGATAAA). Forward and reverse oligonucleotides (Life Technologies) were annealed and cloned into the pLVTHM lentiviral vector (Addgene 12247). Virus was produced in 293T cells after the CaCl_2_-mediated transient transfection of the lentiviral constructs and the packaging plasmids pMD2.G and psPAX-2 (Addgene 12259 and 12260, respectively). Forty-eight hours after the transfection, the lentiviral supernatants were harvested, clarified by filtration, and immediately used to transduce 5 × 10^6^ primary CLL or MEC-1 cells. At 72 h post-infection, GFP-positive cells were sorted on a FACSVantage cytofluorimeter (BD Biosciences) to analyze PCD.

### Animals

Mice were housed at the Bichat Medical School animal facility in strictly controlled, specific-pathogen-free conditions. All experiments were performed in accordance with ARRIVE ethical guidelines [46] and with the approval of the French national committee on animal experimentation (A74–18–01 and 7413). Unless otherwise specified, eight mice (Charles River Laboratories) were used for each experiment/condition.

In the CLL-xenograft model, MEC-1 cells (3 × 10^6^) were injected subcutaneously into NOD/scid gamma (NSG) mice. When the tumor volume reached 100 mm^3^, mice received intraperitoneal injections of PBS, PKHB1, or 4N1K (200 μg in 200 μl of PBS) once a week. Tumor size was measured with a caliper, and tumor volume was calculated using the formula (length × width^2^)/2 and expressed in cubic millimeters. Alternatively, at 28 d after the engraftment, XenoLight RediJect 2-DG 750 Probe (Caliper) was injected into the retro-orbital sinus to visualize tumor glucose uptake, which reflects cell proliferation [47]. Fluorescence was measured using an *in vivo* imaging system FX Pro (Kodak), and pictures were analyzed with Carestream Molecular Imaging software.

To determine hemoglobin levels, tumors were excised from euthanized mice, weighed, and homogenized in PBS. After centrifugation, formic acid was added to the supernatant, and the hemoglobin concentration was calculated based on the absorbance at 405 nm.

To assess PCD, calreticulin exposure, and PLCγ1-Y783, dissociated tumors were digested in medium containing Liberase-TL (Roche), DNase-I (Calbiochem), and Collagenase-IV (Life Technologies). Single-cell suspension, obtained by filtering through a 70-μM cell strainer, was analyzed by flow cytometry.

In mice toxicity studies, we used the same injection protocol as in xenografted NSG mice. The hematological parameters were monitored in blood drawn from the retro-orbital plexus from either vehicle- (PBS) or PKHB1-treated wild-type mice (C57BL/6) with an MS9–5 analyzer (Melet Schloesing). Kidneys and livers (24 h after the third injection of PBS or PKHB1) were fixed and embedded in paraffin. Paraffin sections (2–3 mm thick) of kidney and liver were deparaffinized and rehydrated before staining with periodic acid—Schiff and hematoxylin-eosin, respectively.

### Immunohistochemistry

Tumor vessels were observed on 5-μm cryo-sections by targeting mouse CD31 protein. In order to avoid unspecific background signal, sections were first pretreated with 0.3% H_2_O_2_ and Avidin/Biotin (Vector labs). Then, sections were incubated with a rat anti—mouse CD31 antibody (clone MEC 13.3; BD Biosciences) followed by a goat F(ab′)_2_ anti-rat IgG(H+L)-Biotin (3052–08; Southern Biotech). mCD31 was visualized with the Vectastain Elite ABC Kit (Vector Labs) plus DAB chromogenic substrate (Interchim). Sections were counterstained with hematoxylin and mounted with ImmunoMount (Thermo Scientific).

### Statistical Analysis

One-way ANOVA, Mann-Whitney tests, and Student’s *t*-tests were performed using GraphPad Prism (GraphPad Software). Unless otherwise noted, our assessments included an equal number of Binet Stage A and Binet Stage B/C CLL patients (Tables 1 and 2).

**Table 1:** Features of CLL patients with clinical Binet Stage A.

Patient Number	*IGHV* Mutational Status	ZAP70	CD38	FISH				Karyotype
				Del 17p	Del 11q	Del 13q	Trisomy 12	
1	M	−	−	−	−	+mono[99/200]	−	nd
2	M	−	−	−	−	−	−	Normal
3	M	−	−	−	Failure	+mono[109/200]/bi[9/200]	+[36/600]	nd
4	M	−	+	−	−	−	+[106/200]	nd
5	UM	+	−	−	−	+mono[24/200]	−	Normal
6	UM	+	−	−	+[163/200]	+mono[38/200]	−	1 abnormality
7	UM	+	−	−	−	−	−	Failure
8	M	−	−	−	−	+bi[175/200]	−	nd
9	M	−	−	−	−	+mono[48/200]	−	Normal
10	M	−	−	−	−	+mono[62/200]/bi[99/200]	−	Normal
11	UM	+	+	−	−	−	−	1 abnormality
12	M	−	−	−	−	+mono[78/200]/bi[23/200]	−	Normal
13	UM	+	−	−	+[79/200]	−	−	Failure
14	M	−	−	−	−	−	−	nd
15	M	−	−	−	−	+mono[194/200]	−	nd
16	M	−	−	−	−	+mono[110/200]	−	Normal
17	M	−	−	−	−	+mono[177/200]	−	1 abnormality
18	M	−	−	−	−	+mono[44/200]/bi[140/200]	−	Normal
19	M	−	−	−	−	−	−	Normal
20	M	+	−	−	−	−	−	nd
21	M	−	−	−	−	+mono[137/200]	−	2 abnormalities
22	M	−	−	−	−	−	−	nd
23	M	−	−	−	−	−	−	Complex
24	M	−	−	−	−	+mono[138/200]	−	nd
25	UM	+	+	+[46/200]	−	+mono[28/200]	−	Complex
26	M	−	−	−	−	−	−	Normal
27	M	−	−	−	−	+mono[174/200]	−	nd
28	M	−	−	−	−	+mono[159/200]	−	nd
29	M	+	+	−	−	+mono[118/200]	+[143/200]	nd
30	M	−	−	+[107/200]	−	+bi[165/200]	−	Complex
31	M	−	Nd	−	−	−	−	nd
32	M	−	−	−	−	+mono[133/200]	−	nd
33	M	−	−	−	−	+mono[102/200]	−	Normal
34	M	−	+	−	−	−	−	nd
35	M	Grey zone	−	−	−	+mono[25/200]	−	nd
36	M	−	−	−	−	+mono[126/200]	−	1 abnormality
37	M	−	−	−	−	+mono[164/200]	−	nd
38	UM	+	−	−	−	−	−	Normal
39	M	−	−	−	−	−	−	Normal
40	UM	+	−	−	−	−	−	Normal
41	M	−	Nd	−	−	+mono[32/200]	−	nd
42	M	−	−	−	−	+mono[147/200]/bi[6/200]	−	1 abnormality
43	M	−	−	−	−	+mono[191/200]	−	Failure
44	M	−	−	−	−	+mono[73/200]/bi[94/200]	−	1 abnormality
45	UM	+	+	−	+[88/200]	+mono[179/200]	−	Complex
46	UM	−	−	−	+[189/200]	+bi[185/200]	−	1 abnormality

*IGHV* mutational status homology ≥ 98%: M, mutated; UM, unmutated. ZAP70 is considered negative when the ratio (*R*) of mean fluorescence of T lymphocytes/mean fluorescence of CLL B lymphocytes ≥ 12. ZAP70 is considered positive if *R* ≤ 10, and “grey zone” if 10 < *R* < 12. CD38 is considered positive when the percentage of positive leukemic cells is ≥30%. FISH: score of interphasic cells with an abnormality. Complex karyotype is ≥3 chromosomal abnormalities. bi, biallelic; Del, deletion; mono, monoallelic; nd, not done.

**Table 2:** Features of CLL patients with clinical Binet Stage B/C.

Patient Number	*IGHV* Mutational Status	ZAP70	CD38	FISH				Karyotype
				Del 17p	Del 11q	Del 13q	Trisomy 12	
47	UM	+	+	−	+[174/200]	+mono[193/200]	−	Normal
48	M	−	−	−	−	−	−	Normal
49	M	−	−	−	+[111/200]	+mono[170/200]	−	nd
50	M	−	−	−	−	+mono[93/200]	−	Failure
51	UM	+	−	−	−	−	−	Failure
52	UM	Grey zone	+	−	+[176/200]	+mono[43/200]	−	1 abnormality
53	UM	−	−	+[178/200]	−	+mono[20/200]	−	Complex
54	UM	+	−	−	+[45/200]	+mono[83/200]	−	Complex
55	M	−	+	−	−	+bi[166/200]	−	Normal
56	M	−	+	−	−	−	−	1 abnormality
57	UM	+	−	−	−	+mono[200/200]	−	1 abnormality
58	M	−	−	−	−	+mono[165/200]	−	1 abnormality
59	M	−	−	+[34/200]	−	+mono[15/200]/bi[171/200]	−	Failure
60	UM	+	−	−	−	+mono[180/200]	−	Normal
61	UM	+	+	−	+[149/200]	+mono[146/200]	−	Complex
62	M	−	−	+[195/200]	−	+mono[193/200]	−	Complex
63	UM	+	+	−	+[181/200]	−	−	1 abnormality
64	UM	+	−	−	−	−	−	Normal
65	M	−	Nd	−	−	+bi[123/200]	+[123/200]	2 abnormalities
66	M	nd	+	−	−	−	−	1 abnormality
67	UM	+	−	+[181/200]	−	−	−	2 abnormalities
68	M	−	−	−	−	+mono[174/200]	−	Normal
69	UM	+	+	−	+[59/200]	+mono[10/200]/bi[55/200]	−	Complex
70	M	−	−	−	−	−	+[125/200]	1 abnormality
71	UM	−	−	+[193/200]	−	−	−	Complex
72	UM	+	+	+[186/200]	−	−	−	Complex
73	M	−	−	+[195/200]	−	+bi[192/200]	−	Complex
74	UM	+	−	+[33/200]	−	−	+[110/200]	Failure
75	M	+	−	−	−	+mono[183/200]	−	nd
76	UM	+	−	−	−	+mono[140/200]	−	Complex
77	UM	−	−	−	+[121/200]	+mono[122/200]	−	Normal
78	UM	+	+	−	+[169/200]	+mono[173/200]	−	2 abnormalities
79	UM	+	−	−	−	−	+[146/200]	nd
80	UM	+	+	−	−	+mono[43/200]	−	Complex

*IGHV* mutational status homology ≥ 98%: M, mutated; UM, unmutated. ZAP70 is considered negative when the ratio (*R*) of mean fluorescence of T lymphocytes/mean fluorescence of CLL B lymphocytes ≥ 12. ZAP70 is considered positive if *R* ≤ 10, and “grey zone” if 10 < *R* < 12. CD38 is considered positive when the percentage of positive leukemic cells is ≥30%. FISH: score of interphasic cells with an abnormality. Complex karyotype is ≥3 chromosomal abnormalities. bi, biallelic; Del, deletion; mono, monoallelic; nd, not done.

## Results

### Human Samples: Demographic and Clinical Characteristics

Peripheral blood samples from CLL patients were provided by the Hematology Department at Pitié-Salpêtrière Hospital. A total of 80 CLL patients were selected for this study, including 29 women and 51 men, with a mean age of 73 ± 11 y (range: 46–96 y). Of these, 46 patients were Stage A and 34 were Stage B or C according to the Binet classification, and 11 of the patients had a *TP53* dysfunction. A more detailed description of the clinical characteristics of the CLL patients is reported in Tables 1–3, including ZAP70 and CD38 expression, cytogenetics, and *IGHV* mutational status. As a control in our experiments, peripheral blood samples from healthy donors were provided by the French Blood Establishment (Etablissement Français du Sang) transfusion center. In our study we included blood samples from 20 healthy donors (age range: 20–70 y) with a gender ratio similar to that of the CLL panel.

**Table 3:** CLL patients with ***TP53*** abnormalities.

Patient Number	Del 17p (FISH)	P53 Functional Assessment	*TP53* Mutational Status
25	23% [46/200]	Dysfunctional	c.536A>G/p.H179R (exon 5)
30	53% [107/200]	Dysfunctional	c.425C>G/p.P142R (exon 5) + c.665C>T/p.P222L (exon 6)
53	89% [178/200]	Dysfunctional	Unmutated (exons 5–8)
59	17% [34/200]	Dysfunctional	c.Del790–792CTA/p.Del264L (exon 8)
62	97% [195/200]	Dysfunctional	c.417G>T/p.V173L (exon 5)
67	90% [181/200]	Dysfunctional	c.524G>A/p.R175H (exon 5)
71	96% [193/200]	Dysfunctional	c.184G>T, p.E62X (exon 4)
72	93% [186/200]	Dysfunctional	p.R273H (exon 8)
73	97% [195/200]	Dysfunctional	c.818G>A, p.R273H (exon 8)
74	16% [33/200]	Dysfunctional	c.536C>T/p.H179Y (exon 5)
79	0% [0/200]	Dysfunctional	IVS9+2A>G (intron 9) (homozygous)

The assessment of the functional status of P53 in CLL cells was based on induction of P21 and P53 protein expression using etoposide and nutlin-3a [39]. The detection of *TP53* mutations was performed as described in Methods. FISH: score of interphasic cells with an abnormality. Del, deletion.

### PKHB1, a Human-Serum-Stable TSP1-Derived Peptide, Selectively Kills Leukemic CD5^+^ B Lymphocytes, including Those from Individuals with Dysfunctional *TP53*

**Fig 1:**
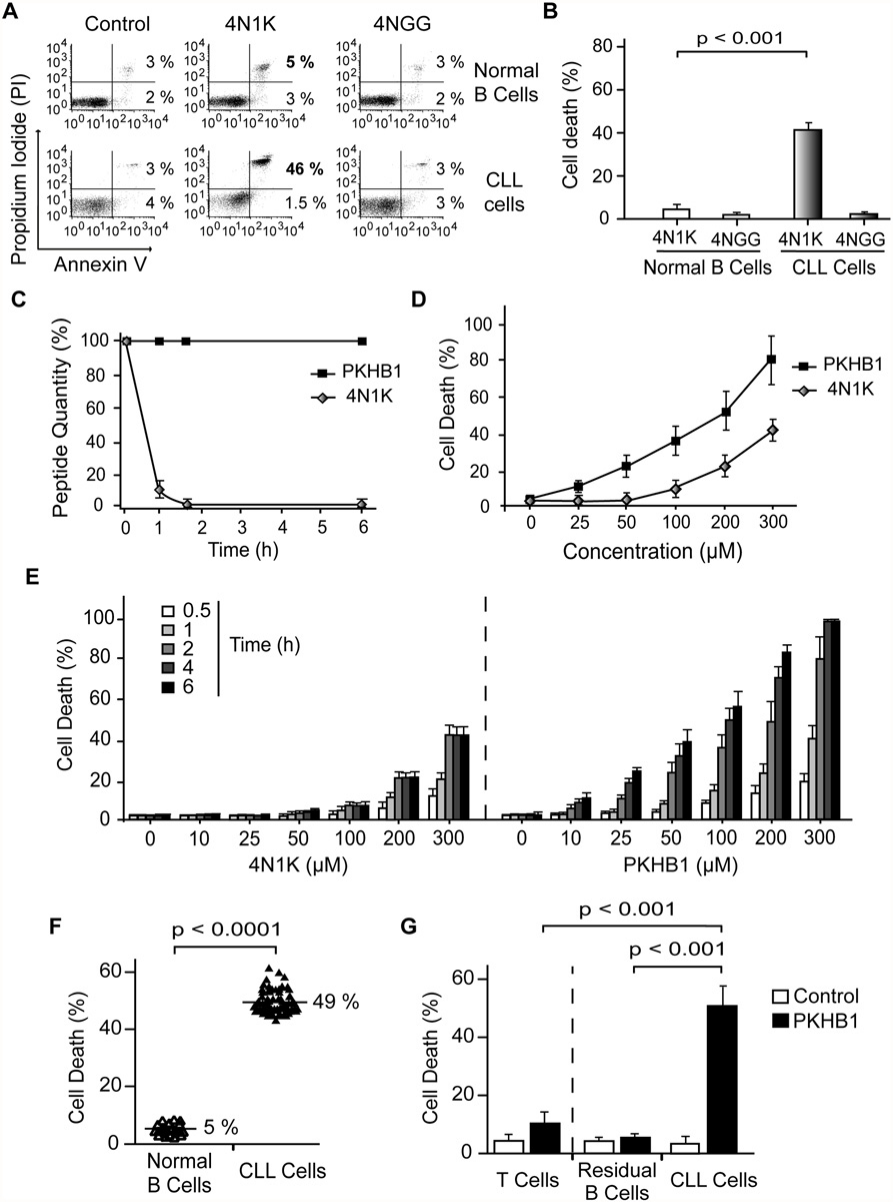
PKHB1 selectively induces cell death in leukemic B cells, including those from patients with dysfunctional *TP53*. (A) Cell viability measured by Annexin-V and PI staining in normal or CLL B lymphocytes treated with 4N1K (300 μM, 2 h) or the negative control peptide 4NGG (300 μM, 2 h). The percentages refer to Annexin-V-positive or Annexin-V-positive/PI-positive staining. (B) B cells from healthy donors (*n* = 8) and CLL patients (*n* = 20) were treated and analyzed as in (A) and graphed. The panel of patients used here included an equal number of CLL patients with Binet Stage A and Binet Stage B/C. Cell death refers to Annexin-V-positive/PI-positive labeling. (C) The proteolytic stabilities of 4N1K and PKHB1 were evaluated in human serum at the indicated times. A mixture of peptide and human plasma was incubated at 37°C, and the kinetics of degradation was followed using HPLC. The relative concentrations of the remaining soluble peptides were analyzed by the integration of the absorbance at 220 nm as a function of retention time. (D) Cell death, measured as Annexin-V and PI co-positivity, was assessed as in (A) in leukemic cells treated with different concentrations of 4N1K or PKHB1. The data in the plot are the mean ± standard deviation (SD) (*n* = 5). (E) After treatment with the indicated concentration of 4N1K or PKHB1 for the indicated time, cell viability was assessed in the CLL cells using Annexin-V-positive/PI-positive staining. The data are graphed as mean ± SD (*n* = 6). (F) Cell death induced by PKHB1 (200 μM, 2 h) was measured in B cells from 20 healthy donors and the cohort of 80 CLL patients described in Tables 1–3. The percentages refer to the mean of the Annexin-V-positive/PI-positive staining. Statistical significance was analyzed with the *t*-test. (G) Cell death was measured as in (F) in PKHB1-treated CD19^−^/CD3^+^ T cells (T cells), CD19^+^/CD5^−^ B cells (residual B cells), and CD19^+^/CD5^+^ B lymphocytes (CLL cells) from CLL patients. The data, which refer to Annexin-V and PI co-positivity, are presented as mean ± SD (residual and leukemic B cells, *n* = 20; T cells, *n* = 8). Unless otherwise indicated, the statistical analyses included in this figure were performed with the Mann-Whitney test.

To explore the potential of CD47 activation by peptide targeting in CLL, we first analyzed the triggering of CD47 with the decapeptide 4N1K. This ligand of CD47 [10,16,29–31,48] induces PCD *in vitro* in breast tumors and leukemic cells [23,34–37]. We corroborated that after only 2 h of treatment with soluble 4N1K (300 μM), 46% of the CLL cells obtained from 20 CLL patients were Annexin-V-positive/PI-positive (Fig. 1A and 1B). In contrast to 4N1K, the negative control analogue 4NGG [35] was ineffective at inducing cytotoxicity in CLL cells. Note that, contrary to B6H12, the more commonly used anti-CD47 mAb [18,23] (S1 Fig), 4N1K induces PCD in CLL cells in soluble conditions. Strikingly, 4N1K incubation had no effect on the normal B lymphocytes isolated from eight healthy donors (Fig. 1B). The Mann-Whitney test substantiated the significance of the different response to 4N1K observed in the malignant and the normal B cells (*p* < 0.001). The specificity of soluble 4N1K to induce PCD in leukemic cells, but not in normal B lymphocytes, led us to further investigate the therapeutic potential of this decapeptide.

The major weakness in the use of peptides as therapeutic agents is their short *in vivo* half-life due to protease degradation. Using an HPLC approach, we observed that a 1-h incubation in human serum resulted in more than 90% of the 4N1K peptide being degraded (Fig. 1C). For that reason, we sought to improve 4N1K stability in human serum by replacing selected natural L amino acids with their D counterparts [49]. The N- and C-terminal lysines of 4N1K, which were introduced to improve solubility, are not related to the CD47 interaction site of the peptide (VVM motif) [50]. Therefore, we replaced these two terminal residues with their D analogues. This novel decapeptide, PKHB1, was not degraded during long-term incubation in human serum (Fig. 1C), but maintained its solubility and the ability to bind CD47 (S2 Fig). This specific binding was validated by the disruption of the PKHB1–CD47 interaction with hSIRPα-Fc (a fusion protein designed to specifically bind CD47 [40,41]), which led to the inhibition of PKHB1-mediated PCD (S3 Fig). Finally, as was expected based on its serum stability and the improved affinity to CD47 (Figs. 1C and S2), PKHB1 induced PCD in the CLL cells more potently than 4N1K did, in terms of both concentration level and incubation times. For example, at 2 h of treatment with 200-μM peptides, PKHB1 induced cytotoxicity in ~49% of the CLL cells, whereas 4N1K induced PCD in only ~25% of the leukemic B cell population (Fig. 1D and 1E).

The PCD response to PKHB1 was verified in 20 B lymphocyte samples from healthy donors and in B cells obtained from the 80 CLL patients described above and in Tables 1 and 2. This CLL cohort incorporated individuals with positive and adverse prognostic features, including those with Binet Stage B and C; unmutated *IGHV*; positive ZAP70 and CD38 expression; 11q, 13q, or 17p deletion; or trisomy 12. We used PKHB1 at 200 μM, a concentration that resulted in PCD responsiveness in CLL cells similar to that of 300-μM 4N1K at 2 h of treatment. With this length of treatment, the spontaneous apoptosis of the primary normal and CLL B cells is less than 5%. As shown in Fig. 1F, the PKHB1-treated CLL cells underwent a rapid cell viability loss (a mean of 49%). Similar to 4N1K, PKHB1 had no effect on the B lymphocytes from healthy donors (a mean of 5% cytotoxicity). The significance of this difference was verified by *t*-test (*p* < 0.001). More interesting from a future therapeutic perspective, 200-μM PKHB1 treatment for 2 h killed the CD5^+^ tumor B cells (~50% of Annexin-V-positive/PI-positive cells) while sparing the residual CD5^−^ B lymphocytes and T cells of the CLL patients (~5% and ~10% cytotoxicity, respectively; Fig. 1G). Using a Mann-Whitney test, we observed that the difference in PCD response to PKHB1 of the malignant B cells and the residual CD5^−^ B lymphocytes and T cells of the CLL patients is highly significant (*p* < 0.001). Overall, these findings suggest that PKHB1 selectively kills the leukemic B cells.

**Fig 2:**
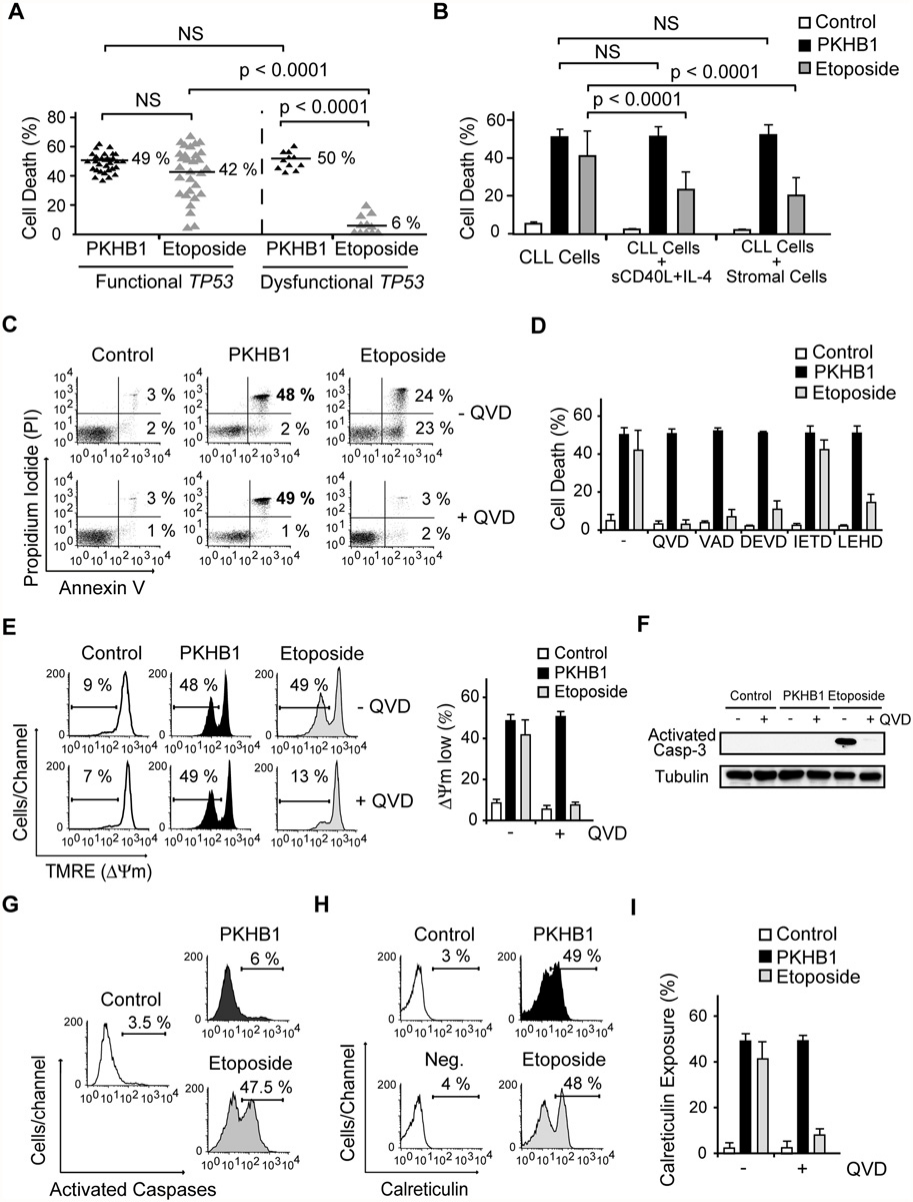
PKHB1 induces calreticulin exposure and caspase-independent PCD in CLL cells. (A) PCD induced by PKHB1 (200 μM, 2 h) or etoposide (250 μM, 12 h) was measured in B cells from 30 CLL patients with functional *TP53* and 11 CLL patients with dysfunctional *TP53*. The percentages refer to Annexin-V-positive/PI-positive staining. (B) Cell death, measured as Annexin-V-positive/PI-positive staining, was detected in CLL cells, CLL cells incubated with sCD40L and IL-4, and CLL cells co-cultured with the bone marrow stromal cell line M210B4 prior to treatment with PKHB1 (200 μM, 2 h) or etoposide (250 μM, 12 h). The data are presented as mean ± SD (*n* = 8 patients). (C) Cell viability was determined in CLL cells that were pre-incubated with vehicle (−) or QVD (+) and were treated with PKHB1 (200 μM, 2 h) or etoposide (250 μM, 12 h). The percentages refer to Annexin-V-positive or Annexin-V-positive/PI-positive staining. (D) PKHB1- or etoposide-mediated cell death was evaluated by Annexin-V and PI staining in CLL cells pretreated with a panel of broad spectrum or specific caspase inhibitors. The data, which refer to Annexin-V-positive or Annexin-V-positive/PI-positive labeling, are graphed as mean ± SD (*n* = 7). (E) ΔΨ_m_ loss was induced by PKHB1 (200 μM, 2 h) or etoposide (250 μM, 12 h) in CLL cells pretreated with vehicle or QVD. The data are plotted as mean ± SD (*n* = 7). (F) A representative immunoblot of activated caspase-3 is shown for the untreated (control) and PKHB1- or etoposide-treated CLL cells pre-incubated with vehicle (−) or QVD (+). Equal loading was confirmed by α-tubulin probing. (G) B lymphocytes from a representative CLL donor were left untreated (control) or were incubated with PKHB1 (200 μM, 2 h) or etoposide (250 μM, 12 h) before assessment of caspase activity with a FAM caspase detection kit. The percentages refer to positive staining. (H) Calreticulin exposure was assessed in untreated (control) and PKHB1- or etoposide-treated CLL cells. The percentages refer to calreticulin-positive cells. Note that, in the absence of α-calreticulin, the control isotype antibody yields negative results (“Neg.” in the cytofluorometric plot). (I) Calreticulin exposure was ascertained in untreated (control) and PKHB1- or etoposide-treated CLL cells in the absence (−) or presence (+) of the caspase inhibitor QVD. The data are presented as mean ± SD (*n* = 9). Statistical analyses in (A) and (B) were performed using one-way ANOVA. NS, not significant.

In order to better characterize the effect of the CD47 agonist peptide PKHB1 in high-risk CLL patients, we compared the response of cells with functional and dysfunctional *TP53* (characterized in S4 Fig and Table 3) to PKHB1 and the P53-dependent PCD inducer etoposide. In cells with functional *TP53*, a 12-h etoposide treatment was required to provoke PCD comparable to that obtained after 2 h of incubation with PKHB1. B lymphocytes with dysfunctional *TP53*, which are resistant to etoposide, were killed after 2 h of incubation with PKHB1 (Fig. 2A). Thus, targeting CD47 with PKHB1 efficiently killed CLL cells, including those from individuals with dysfunctional *TP53*.

Next, given that the microenvironment plays a critical role in the progression and drug resistance of tumors [51,52], we analyzed whether PKHB1-mediated PCD is modulated by the presence of either bone marrow stromal cells or sCD40L and IL-4, two anti-apoptotic cytokines that are generated by lymphoid tissues. Under these conditions, we observed that the responsiveness of CLL cells to PKHB1 remained unchanged (Fig. 2B). In contrast, as corroborated by one-way ANOVA (*p* < 0.001), the induction of PCD by etoposide was significantly diminished. These data strongly suggest that, contrary to other forms of cell death, PKHB1-mediated cell death is not down-regulated by the survival stimuli provided by the lymphocyte microenvironment.

### PKHB1 Induces Caspase-Independent Programmed Cell Death in Chronic Lymphocytic Leukemia Cells

We next assessed the mechanism regulating PKHB1-induced PCD in CLL cells. In contrast to etoposide-induced caspase-dependent PCD, the features of PKHB1-induced killing—including phosphatidylserine exposure, cell viability loss, and ΔΨ_m_ disruption—were not prevented by pre-incubation with broad spectrum or specific caspase inhibitors (Fig. 2C–E). The main effector caspase-3 remained an inactive pro-enzyme after the CLL cells were treated with PKHB1 (Fig. 2F). Thus, it seems that this CD47 peptide agonist induces caspase-independent PCD. This result was substantiated with the help of a FAM-labeled analogue that binds to the active site of the caspases. In contrast to etoposide triggering of cell death (~47% of positive cells), after PKHB1 triggering, the leukemic CLL cells displayed low caspase labeling (6% of positive cells) (Fig. 2G). Finally, as shown in Fig. 2H and 2I, PKHB1 also provoked a caspase-independent exposure of calreticulin, a protein that enables phagocytes to efficiently engulf dead cells [53]. Note that, in contrast to the constitutive expression of calreticulin in acute leukemia cells and solid tumors [54], this protein was not detected on the surface of untreated CLL cells (“control” in Fig. 2H). Thus, PKHB1 activated a caspase-independent PCD path that provoked calreticulin exposure on the surface of the dying cells. *In vivo*, such exposure would be expected to enable the dying cells to be recognized and engulfed [53].

### PKHB1 Provoked Programmed Cell Death in Chronic Lymphocytic Leukemia Cells via Endoplasmic Reticulum Stress, Ca^2+^ Overload, and Mitochondrial Damage

**Fig 3:**
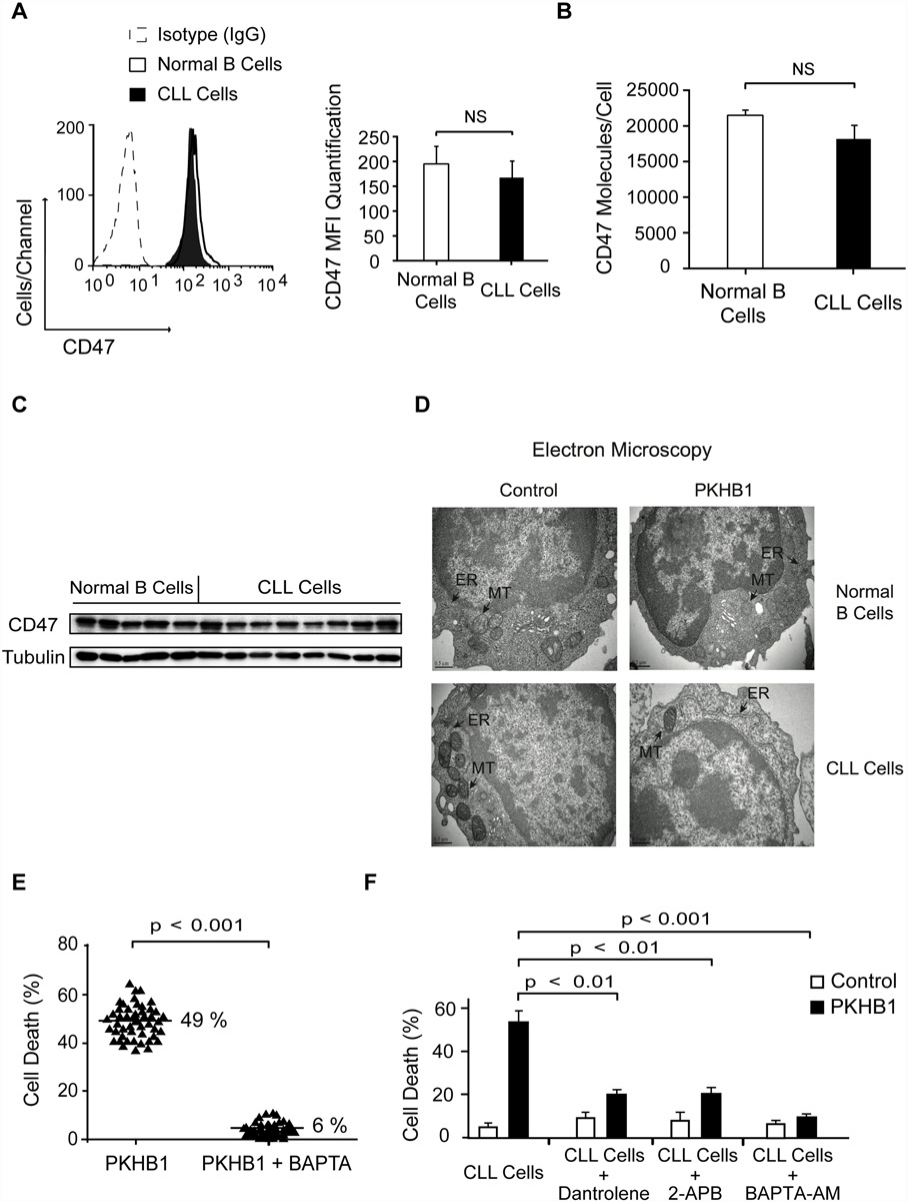
PKHB1 generates endoplasmic reticulum stress that provokes Ca^2+^-mediated PCD in the CLL cells. (A) CD47 expression was quantified using flow cytometry in normal and CLL B cells. A representative cytofluorometric plot is presented. In the bar chart, CD47 was quantified based on the MFI in each sample. The data are presented as mean ± SD (normal B cells, *n* = 20 healthy donors; CLL cells, *n* = 50 patients). (B) The cell surface expression of CD47 was quantified using a QuantiBRITE flow cytometry system. The number of CD47 molecules/cell in normal (*n* = 5 healthy donors) and CLL (*n* = 12 patients) B cells is plotted. (C) CD47 expression in normal and CLL B lymphocytes was determined by immunoblot analysis. Equal loading was confirmed by α-tubulin probing. (D) Representative electron micrographs of untreated (control) or 200-μM PKHB1-treated normal and CLL B cells (2 h of treatment). The black arrows denote the endoplasmic reticulum (ER) and the mitochondria (MT). Bar: 0.5 μm. (E) Cell death was measured in PKHB1-treated B cells (200 μM, 2 h) from 50 CLL patients pre-incubated with vehicle or the external Ca^2+^ chelator BAPTA. The percentages refer to the mean of the Annexin-V-positive/PI-positive staining. (F) Cell death was measured by Annexin-V-positive/PI-positive labeling in PKHB1-treated CLL cells (200 μM, 2 h) pre-incubated with vehicle, dantrolene, 2-APB, or BAPTA-AM. The data are plotted as mean ± SD (*n* = 10). Statistical relevance was assessed with the Mann-Whitney test in (A) and (B) and the *t*-test in (E) and (F).NS, not significant.

From the above experiments, we learned that PKHB1 treatment triggers PCD in leukemic but not in normal B lymphocytes. Interestingly, using flow cytometry and immunoblot analyses, we did not see a correlation between PCD response to PKHB1 and the level of CD47 expression on the cell surface of the malignant and normal B cells (Fig. 3A–C). Therefore, we searched for the specific PCD mechanism that could be activated by PKHB1 in CLL cells, but not in normal B lymphocytes.

The absence of caspase activation observed after CD47 peptide triggering of cell death led us to evaluate the potential role of serpases, the F-actin cytoskeleton, and the fission protein DRP1 in PKHB1-mediated PCD in CLL cells. These three elements are key in the regulation of the caspase-independent cell death process induced in CLL cells by the immobilized anti-CD47 mAb B6H12 [24,55,56]. Using a fluorochrome-labeled analogue, we corroborated that, after PKHB1 triggering, the leukemic CLL cells activated the serpase family of proteases (~45% of cells showed positive serpase labeling) (S5A Fig). To analyze the F-actin cytoskeleton, we measured the intracellular F-actin/G-actin ratio (fibrillar actin = polymerized actin; globular actin = depolymerized actin) [57] in PKHB1-treated cells. This approach showed that, similar to the immobilized anti-CD47 mAb B6H12, PKHB1 ligation provoked actin depolymerization. Interestingly, inhibition of the serpases by TPCK controlled actin damage (S5B Fig). These data suggest a hierarchical relationship between serpases and F-actin depolymerization in PKHB1-mediated PCD. Surprisingly, in contrast to the immobilized anti-CD47 mAb [55], the presence of the cell death effector DRP1 was not observed in the mitochondria of PKHB1-treated CLL cells (S5C Fig). Thus, it seems that PKHB1 and the immobilized anti-CD47 mAb B6H12 induce different types of caspase-independent killing in the CLL cells.

Because morphological alterations in intracellular organelles correlate with the different forms of PCD [58], we next performed an ultra-structural analysis of PKHB1-treated CLL cells. We observed that PKHB1 treatment induced a significant dilation of the endoplasmic reticulum (ER) in leukemic CLL cells, but not in normal B lymphocytes. Moreover, in contrast to immobilized anti-CD47 mAb [55], no morphological changes or swelling were detected in the mitochondria of PKHB1-treated CLL cells (Fig. 3D). Since the ER plays a key role in modulating Ca^2+^ mobilization [59], we considered a potential role of Ca^2+^ in PKHB1-mediated PCD. This role was confirmed by pretreating CLL cells with the Ca^2+^ chelator BAPTA prior to incubation with PKHB1. As depicted in Figs. 3E and S5, this pretreatment abolished the features characterizing PKHB1-mediated killing. A similar result was obtained by incubating CLL cells in a Ca^2+^-free medium prior to treatment with PKHB1 (S5D Fig). Moreover, as corroborated by a *t*-test, pre-incubation with BAPTA-AM (intracellular Ca^2+^ chelator; *p <* 0.001), 2-APB (inositol-1,4,5-triphosphate receptor [IP_3_R] inhibitor; *p <* 0.01), or dantrolene (ryanodine receptor inhibitor; *p <* 0.01) significantly decreased PKHB1-mediated death (Fig. 3F). Altogether, these findings suggest that PKHB1 treatment induces ER stress, which provokes Ca^2+^ overload and PCD in CLL cells.

**Fig 4:**
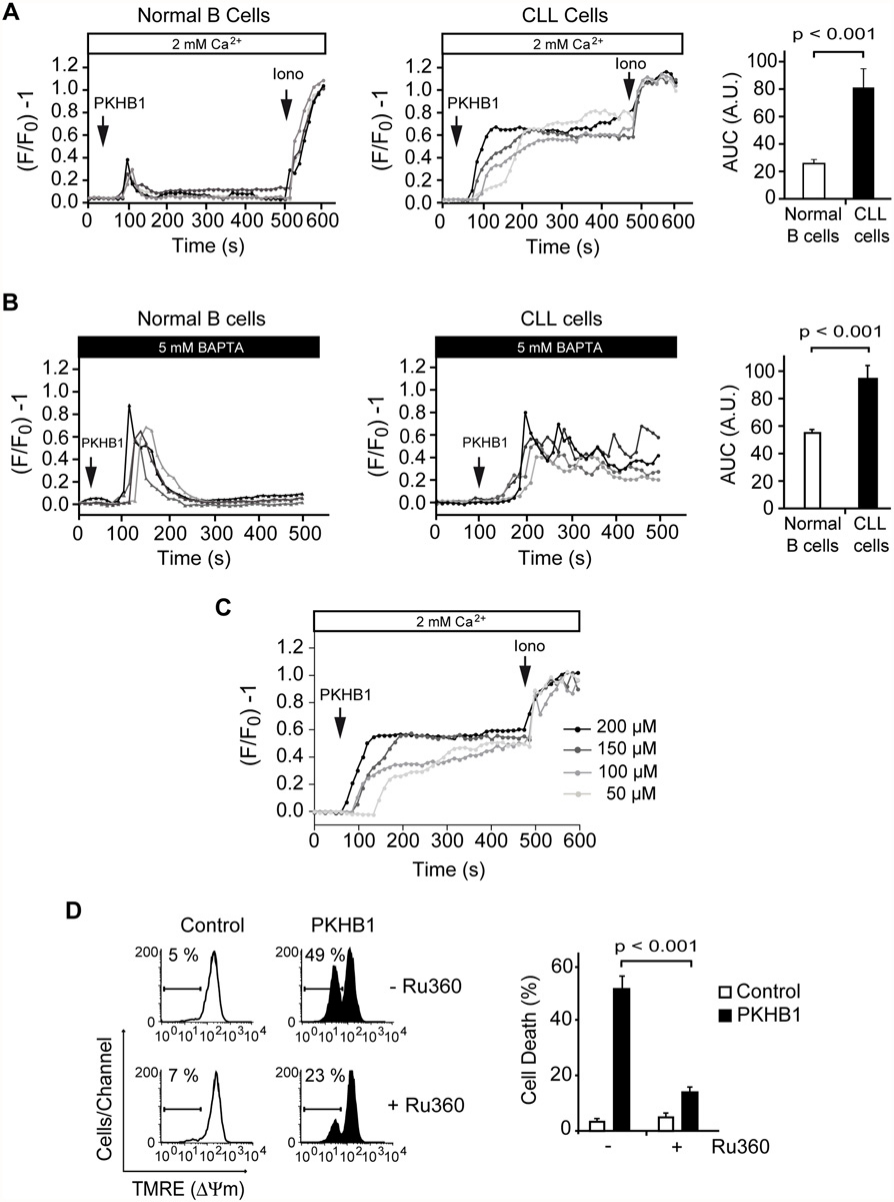
PKHB1 treatment results in sustained Ca^2+^ mobilization that induces mitochondrial damage and PCD in CLL cells. (A) Representative Ca^2+^ mobilization recorded in 200-μM PKHB1-treated normal (left) and CLL (right) B cells (*n* = 4 each). Ionomycin (Iono, 1 μM) was utilized as a control to show the maximum response. The histograms show mean ± SD of the area under the curve (AUC) (in arbitrary units [A.U.]) (*n* = 12). (B) Representative Ca^2+^ release from internal stores was visualized in PKHB1-treated normal (left) and CLL (right) B cells (*n* = 4 each) in Ca^2+^-free medium (plus 5 mM BAPTA). Histograms represent mean ± SD of the area under the curve (*n* = 13). (C) Representative Ca^2+^ mobilization curves were recorded in CLL cells after treatment with different concentrations of PKHB1. Ionomycin (1 μM) was used as a control to show the maximum response. (D) The loss of ΔΨ_m_ induced by PKHB1 (200 μM, 2 h) was measured in CLL cells pre-incubated with vehicle (−) or the mitochondrial Ca^2+^ uniporter inhibitor Ru360 (+). Representative cytofluorometric plots are shown. The percentages refer to cells with low ΔΨ_m_. The data from eight patients are presented in a plot as mean ± SD. The Mann-Whitney test was used in (A) and (B) and the *t*-test in (D).

Next, we compared Ca^2+^ mobilization in normal and CLL B lymphocytes using a fluorescence video-microscopy technology that analyzes the Ca^2+^ signal in single cells. In normal B cells, PKHB1 triggered a classical Ca^2+^ signal with a rapid transient increase in intracellular Ca^2+^ that then returned to baseline [59,60] (Fig. 4A, left panel, and S6A Fig, upper panels). In CLL cells, PKHB1 incubation triggered a strong and sustained Ca^2+^ mobilization that did not return to basal level (Fig. 4A, right panel, and S6A Fig, lower panels). Thus, PKHB1 seems to provoke a different Ca^2+^ mobilization in normal and leukemic cells. To determine whether the sustained Ca^2+^ mobilization observed in PKHB1-treated CLL cells was the consequence of a sustained Ca^2+^ influx or of persistent Ca^2+^ release from intracellular stores, we analyzed the Ca^2+^ mobilization generated by PKHB1 in cells incubated in a Ca^2+^-free medium. In PKHB1-treated normal B lymphocytes, Ca^2+^ released from internal stores returned to basal levels. However, in CLL cells, PKHB1 incubation triggered a sustained Ca^2+^ release from internal stores, which did not return to basal level (Figs. 4B and S6B). Overall, these data indicate that the Ca^2+^ overload observed in PKHB1-treated CLL cells appears to be the consequence of continuous liberation of Ca^2+^ from the ER. Moreover, Ca^2+^ mobilization and PCD increased in PKHB1-treated CLL cells in a dose-dependent manner (Figs. 4C and 1D). These results strongly support a direct link between Ca^2+^ mobilization and induction of PKHB1-mediated PCD in CLL.

An important question arising from our above data is how the PKHB1-induced sustained Ca^2+^ mobilization affects CLL cell viability. Because regulated Ca^2+^ entry into the mitochondria is required to maintain intracellular Ca^2+^ homeostasis [61], we analyzed whether the sustained Ca^2+^ mobilization induced by PKHB1 treatment in CLL cells affected the mitochondria. As shown in Fig. 4D, a *t*-test analysis indicated that the pharmacological blockade of Ca^2+^ entry into the mitochondria using the mitochondrial Ca^2+^ uniporter inhibitor Ru360 significantly moderated the ΔΨ_m_ loss induced by PKHB1 (49% of cells showed ΔΨ_m_ loss in Ru360-untreated cells, whereas only 23% of cells pre-incubated with Ru360 presented ΔΨ_m_ loss; *p <* 0.001), thus preventing PCD. Therefore, it seems that treatment of CLL cells with PKHB1 induces a Ca^2+^ overload that provokes PCD via mitochondrial damage.

### PLCγ1 Is Over-Expressed in Chronic Lymphocytic Leukemia Cells, with Increased Expression Correlated to Disease Progression

**Fig 5:**
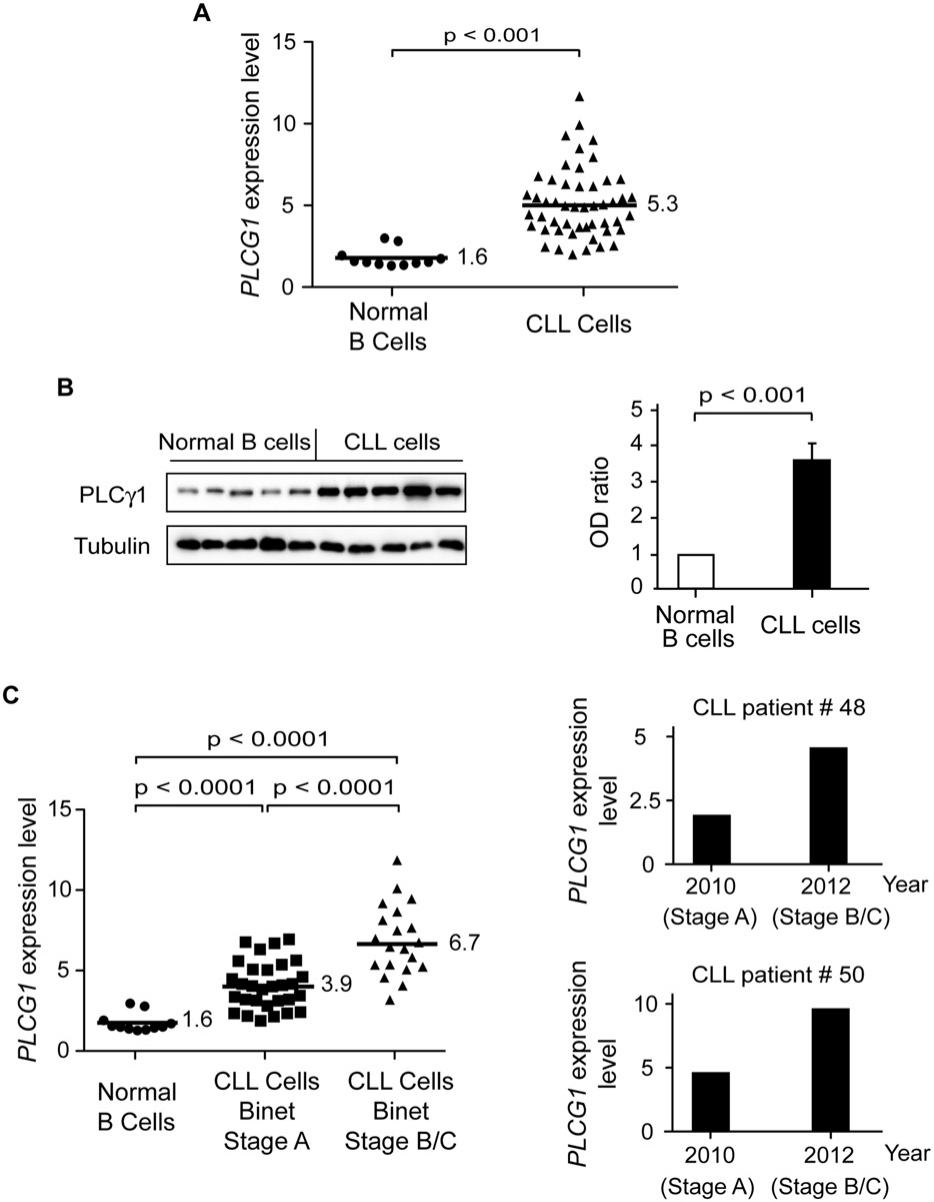
PLCγ1 is over-expressed in CLL cells. (A) *PLCG1* mRNA levels were determined in normal (*n* = 11) and CLL (*n* = 50) B cells. The numbers refer to mean *PLCG1* transcript expression. *GUSB* mRNA expression was used to normalize the data. (B) PLCγ1 was detected by immunoblot analysis in normal and CLL B lymphocytes. Equal loading was confirmed by α-tubulin detection. The optical density (OD) ratio represents the difference in protein expression. The plot depicts mean ± SD (*n* = 3 independent blots). (C) Left: *PLCG1* mRNA levels measured in the normal and leukemic B cells used in (A) are shown by the clinical Binet stage of the CLL patients. The numbers refer to mean *PLCG1* mRNA expression. Right: *PLCG1* mRNA levels measured in patients #48 and #50 at different times. In 2010, both CLL patients were classified as having Binet Stage A, and in 2012 as having Binet Stage B/C. Note that the *PLCG1* mRNA levels measured in these patients correlate with CLL progression. *GUSB* mRNA expression was used to normalize the data. The *t*-test was used in (A) and (C), and the Mann-Whitney test was used in (B).

In order to unravel the molecular mechanism responsible for the sustained Ca^2+^ mobilization leading to PCD in CLL cells—but not in normal B lymphocytes—treated with PKHB1, we performed a quantitative RT-PCR analysis in normal (*n* = 11) and CLL (*n* = 50) B cells of the major genes involved in the regulation of cellular Ca^2+^ homeostasis [59]. Out of the 17 different genes tested, only *PLCG1* mRNA was found to be over-expressed by more than 3-fold in CLL cells compared to normal B lymphocytes (Fig. 5A; Table 4). This difference in expression is highly significant, as verified by *t*-test (*p <* 0.001). The immunoblot assessment depicted in Fig. 5B corroborated the different expression of PLCγ1 in normal and CLL B cells. Because the panel of *PLCG1* mRNA expression in CLL cells was quite scattered (Fig. 5A), we wondered whether there was a correlation between the level of *PLCG1* mRNA and progression of the disease. The statistical *t*-test performed in 50 individuals with CLL indicated, with *p <* 0.001, that more *PLCG1* mRNA is expressed in patients with advanced disease (Binet Stage B/C) than in patients with indolent CLL (Binet Stage A) (Fig. 5C, left panel). This finding is further supported by results obtained in B cells from patients with unfavorable CLL evolution. In these patients, *PLCG1* mRNA was found to be over-expressed in CLL cells obtained at Binet Stage B/C of diagnosis, compared to CLL B lymphocytes purified when the patient was diagnosed at Binet Stage A (Fig. 5C, right panels). Altogether, these data indicate that the expression of *PLCG1* mRNA could be considered a marker of CLL severity.

**Table 4:** Transcript expression levels of Ca^**2+**^-related signaling molecules.

Endogenous Reference	mRNA	Expression in CLL versus Normal B Cells
***GUSB***	*PLCB1*	0.6
	*PLCB2*	1.5
	*PLCB3*	1.2
	*PLCB4*	1.0
	***PLCG1***	**3.3**
	*PLCG2*	1.0
	*ITPR1*	0.5
	*ITPR2*	1.3
	*ITPR3*	0.9
	*RYR1*	1.0
	*RYR2*	1.0
	*RYR3*	1.0
	*STIM1*	1.0
	*STIM2*	2.0
	*Orai1*	1.0
	*Orai2*	1.1
	*Orai3*	1.1
***ABL***	*PLCB1*	1.3
	*PLCB2*	2.2
	*PLCB3*	1.0
	*PLCB4*	1.0
	***PLCG1***	**3.5**
	*PLCG2*	1.7
	*ITPR1*	1.2
	*ITPR2*	1.5
	*ITPR3*	1.5
	*RYR1*	1.0
	*RYR2*	1.0
	*RYR3*	1.0
	*STIM1*	1.6
	*STIM2*	2.2
	*Orai1*	1.3
	*Orai2*	1.7
	*Orai3*	1.7

Quantitative RT-PCR was performed on B cells from 11 healthy donors and 50 representative CLL patients (Binet Stage A, *n* = 30; Binet Stage B/C, *n* = 20; five samples with dysfunctional *TP53*). Expression values indicate the mean value obtained for transcripts in CLL cells related to the mean value obtained for the same transcripts in normal B lymphocytes. Data were analyzed using the comparative threshold cycle method. The amount of cDNA measured is normalized to the endogenous references *GUSB* or *ABL* (see Methods for gene details). *PLCG1* is given in bold.

### The Sustained Activation of PLCγ1 Controls PKHB1-Mediated Programmed Cell Death in Chronic Lymphocytic Leukemia Cells

**Fig 6:**
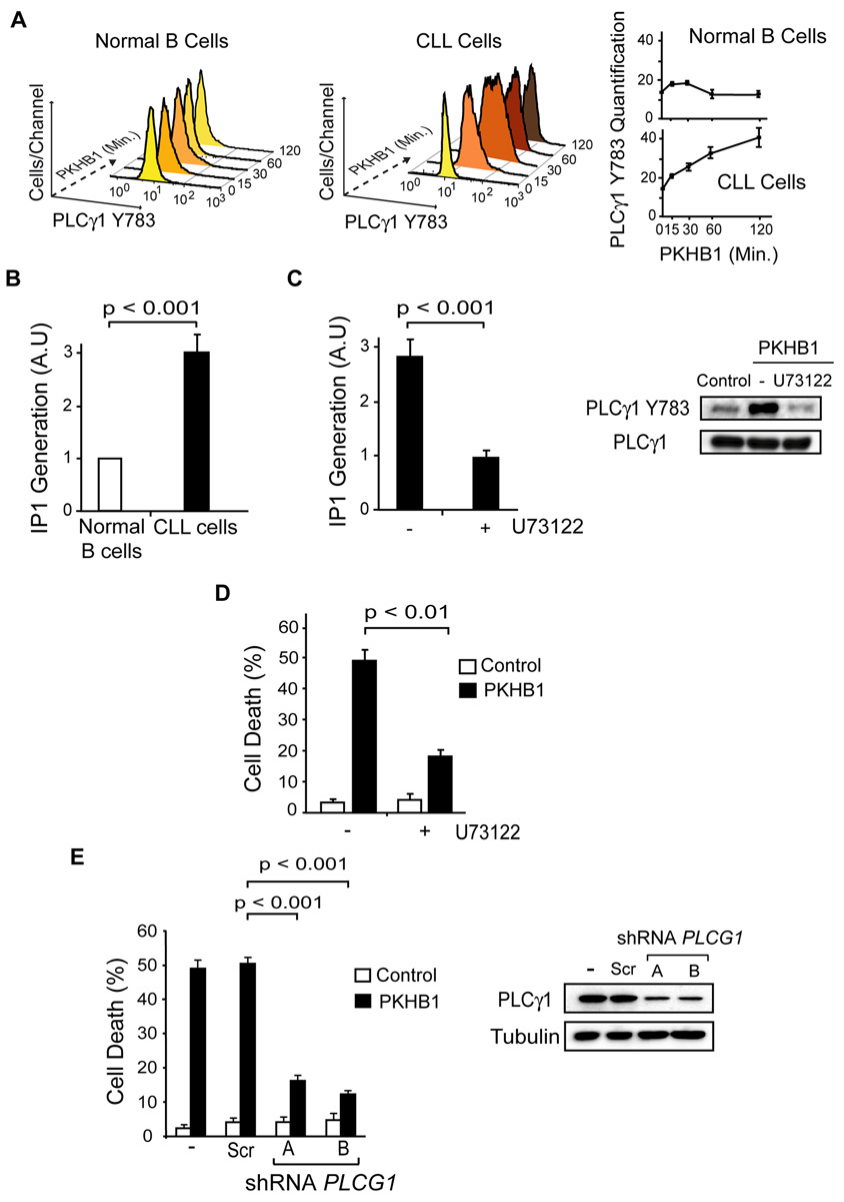
PLCγ1 controls PKHB1-induced PCD. (A) PLCγ1-Y783 phosphorylation was detected at different times in the 200-μM PKHB1-treated normal and CLL B lymphocytes. Representative flow cytometry histograms are shown. In the right panel, the plotted data are presented as MFI ± SD (*n* = 4). (B) IP_1_ was quantified in 200-μM PKHB1-treated normal and CLL B cells at 2 h. The histogram depicts mean ± SD (*n* = 7). (C) IP_1_ generation was determined in 200-μM PKHB1-treated CLL B lymphocytes pre-incubated with vehicle or the PLC inhibitor U73122. The data in the plot are mean ± SD (*n* = 5). Immunoblot analysis confirmed that U73122 inhibited PLCγ1-Y783 phosphorylation in PKHB1-treated cells (200 μM, 2 h). Equal loading was confirmed by PLCγ1 probing. (D) Cell death was measured by Annexin-V-positive/PI-positive staining in untreated (control) and PKHB1-treated CLL cells (200 μM, 2 h) pre-incubated with vehicle or U73122. The data are presented as mean ± SD (*n* = 10). (E) The effects of *PLCG1* down-regulation on CD47-mediated PCD were assessed in PKHB1-treated CLL B cells (200 μM, 2 h) transduced with scrambled shRNA (Scr) or two shRNAs targeting *PLCG1* (shRNA A and B). Cell death, measured by Annexin-V-positive/PI-positive staining, was plotted as mean ± SD (*n* = 6). Changes in PLCγ1 expression were observed by immunoblot analysis. Equal loading was confirmed by α-tubulin detection. Statistical analyses were performed with the Mann-Whitney test in (B) and (E) and the *t*-test in (C) and (D).

PLCγ1 catalyzes the formation of IP_3_, which binds to its receptors on the ER and, subsequently, triggers store-operated Ca^2+^ release. PLCγ1 is over-expressed in CLL; therefore, the disparate Ca^2+^ mobilization recorded in the PKHB1-treated normal and CLL B cells could be related to the differential activation of PLCγ1. Measuring PLCγ1 activation by its phosphorylation at Y783 [62], we observed that PLCγ1 was rapidly phosphorylated before returning to basal levels in the PKHB1-treated normal B cells. However, PLCγ1-Y783 phosphorylation in the PKHB1-treated CLL cells remained high for at least 2 h (Fig. 6A). Note that the kinetics of PLCγ1 phosphorylation matched the kinetics of Ca^2+^ response measured in the normal and CLL B cells (Fig. 4A and 4B). Next, by measuring IP_1_ production in the PKHB1-treated CLL cells, we validated that the sustained PLCγ1-Y783 phosphorylation correlated with the increased catalytic activity of the protein in the CLL B cells (Fig. 6B). Moreover, IP_1_ production following PKHB1 incubation was reduced using the PLC inhibitor U73122 (Fig. 6C), emphasizing the correlation between the over-activation of PLCγ1 and the Ca^2+^ overload measured in CLL cells. Consequently, as indicated by *t*-test (*p <* 0.01), the pre-incubation of CLL cells with U73122 prior to PKHB1 treatment significantly decreased PCD (a mean of ~50% Annexin-V/PI co-positivity was measured in U73122-untreated cells and a mean of ~18% in B lymphocytes pre-incubated with U73122) (Fig. 6D).

In addition to the above pharmacological approach, we assessed the role of PLCγ1 in PKHB1-mediated PCD in primary CLL cells using lentiviral down-regulation of *PLCG1* with two independent shRNAs. As shown in Fig. 6E, the Mann-Whitney test (*p <* 0.001) indicated that the down-regulation of *PLCG1* significantly diminished PKHB1-induced PCD in the primary CLL lymphocytes (~50% Annexin/PI co-positivity in control or scramble-transducted cells and less than 20% in B lymphocytes transducted with a shRNA *PLCG1*). Overall, these findings strongly support the key role of PLCγ1 in the PCD mediated by CD47 peptide targeting in CLL cells.

### Treatment with PKHB1 Reduced Chronic Lymphocytic Leukemia Tumor Burden *In Vivo*

**Fig 7:**
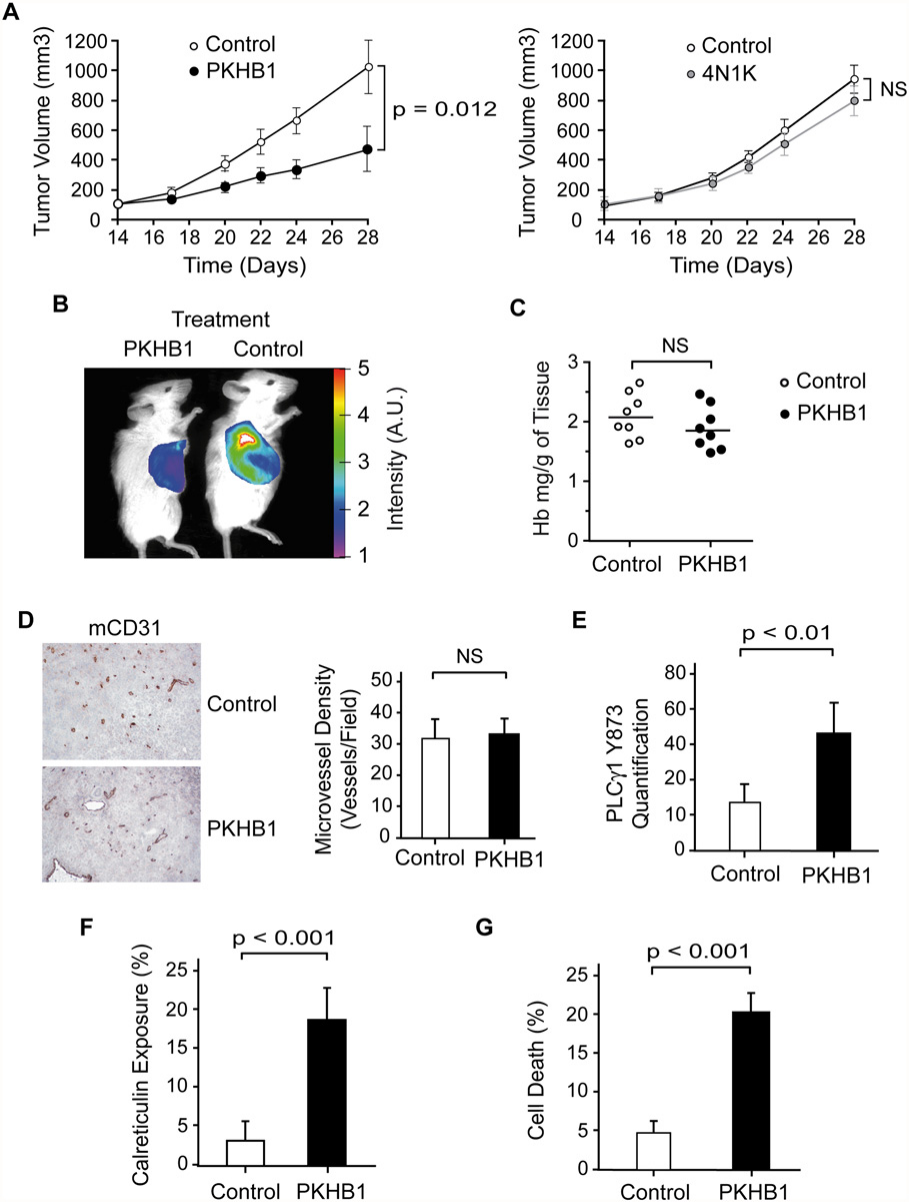
PKHB1 reduced *in vivo* CLL tumor burden by inducing PLCγ1 activation and PCD. (A) NSG mice were subcutaneously transplanted with MEC-1 cells. Starting 14 d after the engraftment, the mice received a weekly intraperitoneal injection of vehicle (control), PKHB1 (left panel), or 4N1K (right panel). Tumor volume was measured using a caliper and graphed. The data are presented as mean ± SD (*n* = 8). In contrast to vehicle or 4N1K treatment, PKHB1 treatment significantly reduced the tumor volume. (B) In an experiment similar to (A), tumor growth was visualized by measuring glucose uptake. The color scale indicates the fluorescence intensity. (C) The hemoglobin (Hb) concentration (*n* = 8 per group) was assessed in vehicle- (control) or PKHB1-treated tumors. The bars indicate the mean of the data obtained. (D) Tumor vascularity was investigated by immunohistochemistry analysis of mCD31. Representative photographs and visual quantification of microvessel density (right panel) confirmed similar vascularity in tumors from vehicle- and PKHB1-treated mice. The data are presented as mean ± SD. (E) PLCγ1-Y783 phosphorylation was assessed in tumors obtained from vehicle- (control) and PKHB1-treated mice. Phospho-PLCγ1 was quantified by flow cytometry using MFI. The data are presented as mean ± SD (*n* = 6). (F) Calreticulin exposure was assessed in tumors from vehicle- (control) and PKHB1-treated mice and graphed. The data are presented as mean ± SD (*n* = 6). (G) Percentage of cell death was measured by Annexin-V-positive/PI-positive staining in tumors obtained from vehicle- (control) and PKHB1-treated mice. The data are plotted as mean ± SD (*n* = 8). The statistical analyses included in this figure were performed with the Mann-Whitney test. A.U., arbitrary units; NS, not significant.

Finally, we analyzed the *in vivo* effect of PKHB1 on the growth of MEC-1 tumors in NSG mice [63]. MEC-1 is an established CLL cell line with dysfunctional *TP53* that is resistant to etoposide treatment but responds to PKHB1 in exactly the same way as primary CLL cells (S7 and S8 Figs.). NSG mice were subcutaneously injected with MEC-1 cells, and the tumors were allowed to grow for 14 d until they reached 100 mm^3^. The mice were then treated intraperitoneally once a week with vehicle or PKHB1. After 2 wk of treatment, the PKHB1-treated, but not the vehicle-treated, mice had a significantly decreased tumor growth rate (~50% tumor volume diminution in PKHB1-treated mice compared to vehicle-treated mice; Fig. 7A, left panel, and Fig. 7B; Mann-Whitney test, *p <* 0.05). However, as could be expected from the instability of the peptide, 4N1K treatment was ineffective (Fig. 7A, right panel). No blood, liver, or kidney toxicity was found in PKHB1-treated mice (S9 Fig). Analysis of the tumors from the PKHB1- and vehicle-treated mice shows similar hemoglobin levels within the engrafted tumors, indicating that PKHB1 did not provoke anemia (Fig. 7C). Moreover, anti-CD31 labeling performed in the tumors from the PKHB1- and vehicle-treated mice suggested that the decreased tumor growth rate induced by PKHB1 was not a consequence of an anti-angiogenic effect (Fig. 7D). Indeed, compared to mice receiving vehicle, the mice receiving PKHB1 showed significantly increased levels of PLCγ1-Y783 phosphorylation (Fig. 7E), as well as enhanced calreticulin exposure (Fig. 7F) and a loss of cell viability (Fig. 7G) within the engrafted tumors (Mann-Whitney test, *p <* 0.001). Together, these findings strongly suggest that treatment with PKHB1 eliminates CLL cells *in vivo* by inducing PLCγ1-mediated PCD.

## Discussion

**Fig 8:**
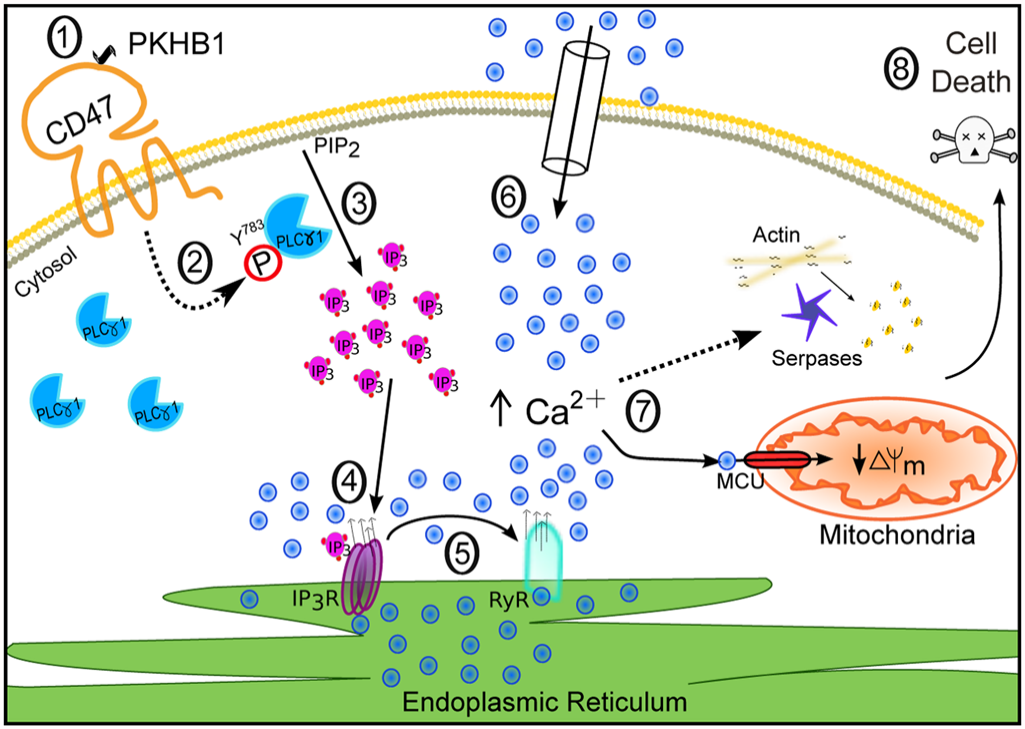
Schematic representation of CD47-mediated PCD in CLL cells. CD47 ligation by the agonist peptide PKHB1 (1) leads to activation of the over-expressed second messenger PLCγ1 by phosphorylation at Y783 (2). PLCγ1-Y783 cleaves phosphatidylinositol 4,5 bisphosphate (PIP_2_) into inositol 1,4,5-trisphosphate (IP_3_) (3), which binds IP_3_Rs in the ER to provoke Ca^2+^ release (4). The increased intracellular Ca^2+^ concentration activates the ER RYRs (5). The decrease of Ca^2+^ in the ER results in the opening of store-operated Ca^2+^ channels in the plasma membrane, provoking substantial calcium entry into the cell (6). This high Ca^2+^ concentration provokes serpase activation, actin depolymerization, and mitochondrial damage (7), followed by PCD (8).

In this work we describe the targeting of CD47 by serum-stable TSP1-derived peptides as a novel approach that could be used to broadly eliminate malignant CLL B cells. We performed *in vitro* cellular and molecular biology assessments in primary CD5^+^ B lymphocytes obtained from a cohort of 80 CLL patients, and we assessed, in a CLL-xenograft mouse model, the *in vivo* capacity of the CD47 agonist peptides to reduce tumor burden. Our *in vitro* approach shows that the CD47 peptide agonists enable a Ca^2+^-mediated, caspase-independent PCD pathway that, sparing the normal T and B lymphocytes, efficiently kills CLL B cells, including those from drug-refractory patients (e.g., with dysfunctional *TP53*). This PCD pathway, to our knowledge molecularly described here for the first time, involves a sequence of events initiated by the triggering of CD47 by serum-stable peptide agonists and the subsequent activation of the signal transduction protein PLCγ1, an over-expressed protein in CLL. Further, PLCγ1 activation leads, by means of the second messenger IP_3_, to ER stress, cellular Ca^2+^ overload, mitochondrial damage, and leukemic B cell death (Fig. 8). The *in vivo* data obtained in the CLL-xenograft mouse model demonstrated that, by inducing PLCγ1-mediated, caspase-independent PCD, the injection of CD47 agonist peptides significantly reduced tumor burden.

### Strategies to Target CD47 for Chronic Lymphocytic Leukemia Treatment

A number of anticancer approaches attempting to kill tumor cells by phagocytosis, antibody-dependent cell-mediated cytotoxicity, or PCD have relied on CD47 targeting by specific mAbs [15,20,64–66]. For CLL, a monoclonal single-chain variable fragment that induces PCD [27] and a therapy that favors the disruption of the CD47–SIRPα link have been proposed [67]. This latter strategy requires the complementary use of rituximab (anti-CD20) to efficiently eliminate tumor cells from a mice xenograft model [67]. In previous research, mainly performed with an immobilized anti-CD47 mAb (B6H12), it has been reported that it is possible to induce PCD *in vitro* in CLL B cells by CD47 triggering [23]. However, as reported in this previous work and confirmed by our current data, when used *in vitro* in soluble conditions, B6H12 is unable to directly induce PCD in CLL B lymphocytes [23] (S1 Fig). With this work in mind, we developed PKHB1, a serum-stable CD47 agonist peptide that, in soluble conditions, induces PCD in malignant CLL B cells. Moreover, we demonstrated that the stabilization of TSP1-derived peptides against serum degradation increases both *in vitro* and *in vivo* biological PCD activity. Overall, our findings strongly support the idea that the targeting of CD47 with peptide agonists could offer medical advantages, such as specificity in provoking PCD in the leukemic CD5^+^ B cells, no detected resistance, and a lack of microenvironment down-regulation. Moreover, as opposed to therapeutic antibodies, peptides and small molecules can be synthesized with a better yield and cost, are less likely to induce immune responses, and do not accumulate in the kidney or liver, thus minimizing toxic side effects [68]. All of these characteristics highlight the viability of the proposed peptide-based approach.

In contrast to the previously described TSP1-derived peptide 4N1K, which induces cell death only *in vitro* [23,34–37], PKHB1 reduces *in vivo* tumor burden in a CLL mouse model. To generate our *in vivo* data, we used one of the few CLL-xenografted mouse models described to date [63], a model widely used in cancer research. Although it does not recreate the physiopathology of human CLL, this CLL-xenograft mouse model fully supports our *in vitro* observations, especially because, contrary to 4N1K, PKHB1 is stable and can reach the xenografted tumor. We also observed in this mouse model that PKHB1 reduced tumor burden by inducing PLCγ1-mediated PCD and that PKHB1 did not induce apparent toxicity in the tumor-engrafted mice.

### Mechanisms of PKHB1-Mediated Cell Death

The targeting of CD47 by peptide agonists enables a form of B cell death that, following the classification of the Nomenclature Committee on Cell Death [69], could be defined as “type III-PCD” or “programmed necrosis.” More precisely, three main hallmarks allow us to classify PKHB1-mediated PCD into the “programmed necrosis” group. First, the killing induced by PKHB1 in the CLL B lymphocytes can be considered “programmed” because it activates the enzymatic machinery of the cell (e.g., by inducing PLCγ1 sustained phosphorylation). Second, PKHB1 enables in the malignant B cells a caspase-independent form of PCD, and not a typical caspase-dependent apoptotic pathway. Finally, the targeting of CD47 by PKHB1 generates biochemical and morphological necrotic features—such as Annexin-V-positive/PI-positive staining and the swelling of intracellular organelles (e.g., the ER) [23,55,69,70]—in the CLL B lymphocytes. As such, PKHB1-mediated cell death shares common features with the mode of PCD induced by the immobilized anti-CD47 mAb B6H12 [23,24,55,56,71]. Certain biochemical features are conserved, namely double Annexin-V-positive/PI-positive staining, activation of the serpase family of proteases, and F-actin network disruption. In addition, both forms of PCD are marked by a dependence on input from several cellular compartments, including the mitochondrion. However, our data suggest that PKHB1-mediated PCD takes place in CLL cells via the induction of cellular alterations that are regulated by the ER and the signal transduction protein PLCγ1, but are independent from the fission protein DRP1, one of the major effectors of killing mediated by anti-CD47 mAb [55]. It seems that, as a consequence of this difference, in contrast to PCD mediated by anti-CD47 mAb, PKHB1 treatment does not provoke morphological changes or swelling in the mitochondria of CLL cells. Therefore, PKHB1-mediated killing and anti-CD47-mAb-induced cell death could represent alternate outcomes of a similar necrotic PCD pathway. The relationship between PKHB1-mediated PCD and other ways to induce PCD by CD47 triggering [27]—which seem to implicate the activation of BNIP3/Hif or the opening of the mitochondrial permeability transition pore—needs to be evaluated in more specific work.

In deciphering how the CD47 agonist peptide triggers PCD, we found that PKHB1 provoked Ca^2+^ overload. The regulation of Ca^2+^ homeostasis within hematopoietic cells is crucial for their function and fate [72]. According to the current model, Ca^2+^ influx is the result of the membrane-receptor-mediated production of IP_3_ following the activation of PLC, which binds to specific receptors on the ER membrane and provokes a quick release of internal Ca^2+^ that is followed by extracellular Ca^2+^ entry through a process known as store-operated Ca^2+^ entry. This Ca^2+^ signaling is transient and controlled, mainly by Ca^2+^ re-sequestration into the mitochondria and ER [73]. Our assessment of the intracellular Ca^2+^ influx after CD47 peptide triggering suggested a totally different pattern of Ca^2+^ mobilization between normal and leukemic B cells. Although the Ca^2+^ influx was rapidly controlled in normal B cells, we recorded a massive and sustained Ca^2+^ mobilization in CLL cells. In these malignant B lymphocytes, the Ca^2+^ overload was due to sustained activation of PLCγ1. This mechanism of PCD induction, described here to our knowledge for the first time in primary B cells, is very similar to that previously reported for Fas-mediated apoptosis in Jurkat T cells [74].

### New Roles for PLCγ1 in Programmed Cell Death and Chronic Lymphocytic Leukemia

Together with other characteristic CLL molecules, such as CD5 or ZAP70, the function of PLCγ1 is mainly associated with the T cell lineage. Indeed, PLCγ1 is critical for T cell function, whereas PLCγ2 is more relevant in platelets, natural killer cells, B cells, and mast cells [75]. In T cells, PLCγ1 is activated downstream of the T cell receptor by a large panoply of SRC, SYK, and TEC kinases. Among them, the SYK-related kinase ZAP70 phosphorylates LAT and SLP76, two adaptors that are critical for PLCγ1 activation. In B cells, cross-linking the BCR results in tyrosine phosphorylation and the activation of PLCγ2 rather than PLCγ1. The SRC, SYK, and TEC kinases also play relevant roles in PLCγ2 activation, which is essential for B cell survival [76]. Related to CLL, IgM—BCR engagement triggers the phosphorylation of SYK, the activation of PLCγ2, and intracellular calcium mobilization [77]. Moreover, some reports have found PLCγ2 to be over-expressed in CLL [78]. In spite of that, our shRNA down-regulation approach indicates that, in PKHB1-mediated PCD, PLCγ1 is the key effector. This original result opens the way to further molecular and cell biology studies. As such, it will be interesting to search for the molecular link between CD47 triggering and PLCγ1-Y783 phosphorylation. As CD47 does not possess tyrosine‐based activation motifs, we hypothesize that the phosphorylation of PLCγ1 after CD47 triggering results from the association of CD47 with other counter-receptors. This association would generate a signalization complex able to (i) activate SRC, SYK, and/or other TEC-related tyrosine kinases and (ii) recruit/phosphorylate PLCγ1. Thus, future work should analyze whether PLCγ1 over-expression plays a role in CLL survival; whether ZAP70 or other SRC, SYK, and TEC kinases are implicated in the sustained PLCγ1-Y783 phosphorylation observed after CD47 peptide triggering; and whether a direct relationship exists between CD47 and BCR or, as in other cell types [79,80], between CD47 and integrins.

Our present study not only highlights a novel PCD role for PLCγ1, but also reveals that *PLCG1* mRNA expression correlates with CLL progression. Even if additional work correlating *PLCG1* mRNA expression level with other CLL prognostic markers (more than 35 have been described to date) is necessary to confirm the clinical value of these findings, our results indicate that *PLCG1* mRNA expression could be a suitable candidate for further consideration as a prognostic marker in CLL.

### Clinical Potential for CD47 Targeting

Deficiencies in the PCD program are frequently involved in tumor drug resistance [81]. The design of novel strategies that bypass this blockade is a major challenge in PCD research. In recent years, most PCD-based pharmacological therapies have focused on the caspase-dependent mode of PCD, also known as apoptosis. In CLL, malignant B cells exhibit alterations that make them resistant to this form of cell death (e.g., ATM/P53 inactivation or over-expression of the anti-apoptotic proteins MCL1 or BCL2). However, there are alternative caspase-independent forms of PCD that could be used to eliminate CLL cells [23,24,55]. Our experiments indicated that the binding of CD47 by the peptide agonists provokes caspase-independent PCD with a broad efficacy (e.g., it is effective even in cells with caspase-dependent PCD blockade). Therefore, the induction of PCD by CD47 peptide targeting overcomes the apoptotic avoidance that is characteristic of CLL.

### Limitations and Strengths

One of the main limitations of our study is the only moderate affinity of the TSP1-derived peptides to CD47. As a consequence, (i) the induction of PCD in primary CLL B lymphocytes was achieved only at a micromolar concentration, still distant from the standard requirements in drug development (nanomolar range), and (ii) the reduction in tumor burden in our CLL-xenograft mouse model was limited. However, based on our original results on peptide serum stability, we may anticipate that a second set of CD47 peptide agonists will be generated with better efficacy and/or bioavailability than PKHB1 (e.g., obtained by introduction of amino acid surrogates). In addition, it is generally agreed that the current CLL animal models do not fully mimic the situation in humans. Our *in vivo* studies were based on one of the previously described CLL-xenografted mouse models [63]. This model involves xenotransplanting MEC-1 cells as a solid mass in NSG mice. Given that CLL is a disseminated disease, our mouse model does not strictly recapitulate CLL features. Consequently, we cannot rule out the possibility that the CD47 peptide agonists might behave differently in CLL patients. Thus, complementary preclinical and toxicological studies in more appropriate animal models (e.g., novel CLL mouse models or primates) will be necessary before the therapeutic effect of PKHB1 and derivatives can be fully assessed in humans. Finally, although PLCγ1 over-expression was clearly observed in CLL B cells, the mRNA assessment of *PLCG1* was done using samples from 50 CLL patients and 11 healthy donors, which is not enough to determine whether this protein could be considered as a prognostic marker in CLL. A more specific study, with a higher number of samples, correlating *PLCG1* mRNA expression level with other CLL prognostic markers will be necessary to verify the clinical value of our findings.

Our work presents several strengths. First, it is a translational study with three parts: (i) generation of PKHB1 (a serum-stable CD47 peptide agonist), (ii) analysis of the biological activity of PKHB1 *in vitro* on a panel of primary B lymphocytes that reproduced the diversity of CLL patients and incorporated the cytogenetic abnormalities that are associated with CLL refractoriness, and (iii) an *in vivo* approach to assess the effect of PKHB1 on a CLL mouse model, which revealed that this peptide reduces tumor burden without apparent side effects. Second, from a mechanistic point of view, our work reveals the existence of a Ca^2+^-mediated, caspase-independent PCD signaling pathway in the tumor cells that could be enabled independently from the classical apoptotic path (e.g., in cells with dysfunctional *TP53*) and that is not down-modulated by the lymphocyte microenvironment. This mode of PCD efficiently and specifically targets malignant cells. Finally, in investigating the molecular determinants regulating PKHB1-mediated killing, we uncovered an unexpected role for PLCγ1 in PCD and revealed a potential link between *PLCG1* mRNA expression and CLL severity. Both within the CLL field and in the broader scope of cancer research, our mechanistic results regarding caspase-independent PCD and PLCγ1 pave the way for the development of novel pharmacological tools that could circumvent the chemotherapy resistance characterizing CLL cells.

## Conclusions

Patients with heavily treated and refractory CLL face a critical medical need that is still unmet. The standard front-line therapy against this leukemia (fludarabine, cyclophosphamide, and rituximab), as well as the current alternative treatments (e.g., anti-CD52, optimized anti-CD20, and anti-CD23 antibodies), can generate refractoriness or undesirable side effects [6]. Recently approved in the United States and Europe, PI3K delta and Bruton tyrosine kinase (BTK) inhibitors represent novel anti-CLL approaches that yield apparent durable remission in patients with relapsed or refractory CLL. Yet, the establishment of PCD approaches such as our peptide-based cell death strategy remains of great interest. Our strategy (i) is less expensive to produce, (ii) is specific to the tumor cells (sparing the residual CD5^-^ B lymphocytes and T cells of the patient), (iii) could be broadly used in CLL patients with high-risk genetic lesions, (iv) is not down-regulated by the lymphocyte microenvironment, and (v) does not provoke apparent anemia or toxicity in a CLL mouse model. Overall, our work represents a first step toward the development of a new peptide-based treatment for CLL, which still remains an incurable disease.

## Supporting Information

S1 Fig Immobilized, but not soluble, CD47 mAb induces PCD in CLL. Cell viability measured in CLL B lymphocytes left untreated or incubated for 2 h with CD47 mAb (5 μg/ml, clone B6H12) in soluble or immobilized conditions. The percentages refer to Annexin-V-positive or Annexin-V-positive/PI-positive staining. The cytofluorometric data from a representative CLL patient are shown. This experiment has been done ten times, yielding low interexperimental variability (<5%).
(TIF)

S2 Fig Primary structure of 4NGG, 4N1K, and PKHB1. In 4NGG, the two central valines of 4N1K were replaced by glycines (highlighted in red). In PKHB1, the N- and C-terminal lysines of 4N1K were replaced with their D counterparts (highlighted in green). The dissociation constants (*K*_d_) of 4NGG, 4N1K, and PKHB1 to CD47—determined by fluorescence polarization, isothermal titration calorimetry, and microscale thermophoresis binding assays [82]—are 1,000,000 nM, 1,500 nM, and 400 nM, respectively.
(TIF)

S3 Fig Disruption of PKHB1–CD47 interaction with the fusion protein hSIRPα-Fc inhibits PKHB1-mediated PCD. Cell viability was determined in CLL cells that were pre-incubated with vehicle (−) or hSIRPα-Fc (+) and were treated with PKHB1 (200 μM, 2 h). The percentages refer to Annexin-V-positive/PI-positive. The data are graphed as mean ± SD (*n* = 5). The statistical analysis included in this figure was performed with the *t*- test.
(TIF)

S4 Fig Flow cytometry analysis of P53 and P21 in CLL B cells. The functional status of *TP53* was determined based on the induction of P53 and P21 protein expression by etoposide in combination with nutlin-3a [39]. The upper panels provide representative cytofluorometric plots of P53 in CLL cells from patient #22 (functional *TP53*, Table 1) and patient #71 (dysfunctional *TP53*, 96% deletion of 17p; Tables 2 and 3). In a similar experiment, the lower panels depict representative plots of P21 expression in B cells from the same individuals as above.
(TIF)

S5 Fig PKHB1-mediated cell death and anti-CD47-mAb-induced PCD represent alternate outcomes of a similar cell death pathway. (A) Left: B lymphocytes from a representative CLL donor were left untreated (control) or were incubated for 2 h with immobilized CD47 mAb (B6H12) or PKHB1 (200 μM) before assessment of serpase activity with a green fluorescent SerPase kit (FFCK). The percentages refer to positive staining. Right: B cells from CLL patients (*n* = 5) were left untreated (control), were incubated with PKHB1 (200 μM, 2 h), or were pre-incubated with the external Ca^2+^ chelator BAPTA prior to PKHB1 treatment. The data are presented as mean ± SD. (B) CLL cells were exposed to vehicle (control), immobilized CD47 mAb (B6H12, 2 h), or PKHB1 (200 μM, 2 h), and the fluorescence F-actin/G-actin ratio was quantified. One unit refers to the basal F-actin/G-actin ratio scored in control cells. Data are the mean of five independent experiments ± SD. (C) B lymphocytes from a representative CLL donor were left untreated (control) or were incubated for 2 h with either immobilized CD47 mAb (B6H12) or PKHB1 (200 μM) before immunoblot detection of DRP1 in the mitochondrial fraction. Equal loading was confirmed by Cox IV probing. (D) Cell death was measured by Annexin-V-positive/PI-positive staining in PKHB1-treated CLL B cells (200 μM, 2 h) pre-incubated with vehicle, the external Ca^2+^ chelator BAPTA, or Ca^2+^-free medium (*n* = 5). The data are graphed as mean ± SD.
(TIF)

S6 Fig Ca^2+^ imaging in PKHB1-treated normal and CLL B cells. Normal B cells from a healthy donor and B lymphocytes from a representative CLL patient were stained with Fura2-AM and pluronic acid in glass bottom dishes. The cells were treated with PKHB1 (200 μM) and imaged using a dual excitation fluorometric imaging system for the indicated time. Cytosolic Ca^2+^ variations were recorded in the presence of 2 mM Ca^2+^ (A) or 5 mM BAPTA (B). The scale bar depicts the relative Ca^2+^ intensity.
(TIF)

S7 Fig PKHB1 induces Ca^2+^-mediated, caspase-independent PCD in MEC-1 cells. (A) MEC-1 cells were left untreated or were incubated for 2 h with PKHB1 (200 μM) or for 12 h with the P53-dependent cell death inducer etoposide (250 μM); the cells were then labeled with Annexin-V and PI to assess cell viability. The percentages refer to Annexin-V-positive or Annexin-V-postsitive/PI-positive staining. (B) MEC-1 cells were incubated as in (A) and stained with TMRE, and ΔΨ_m_ was determined by flow cytometry. The percentages refer to cells with ΔΨ_m_ loss. As depicted in (A) and (B), the dysfunctional *TP53* MEC-1 cell line was resistant to etoposide (12 h of treatment). (C) Cell death was measured by Annexin-V-positive/PI-positive labeling in untreated (control) or PKHB1-treated MEC-1 cells (200 μM, 2 h) pre-incubated with vehicle (−) or the ER receptor inhibitors dantrolene or 2-APB. The data are presented as mean ± SD (*n* = 6). (D) As described in (C), cell death was measured in untreated (control) or 200-μM PKHB1-treated MEC-1 cells pre-incubated with vehicle (−) or the intracellular Ca^2+^ chelator BAPTA-AM. The data, which refer to Annexin-V and PI co-positivity, are mean ± SD (*n* = 5). (E) A representative Ca^2+^ mobilization triggered by 200 μM PKHB1 in MEC-1 cells is illustrated. Ionomycin (Iono, 1 μM) was used as a control to demonstrate the maximum response. The data are presented as mean ± SD (*n* = 26 cells). The statistical analysis included in this figure was performed with the *t*-test.
(TIF)

S8 Fig PLCγ1-Y783 phosphorylation controls PKHB1-induced killing in MEC-1 cells. (A) At the indicated time, PLCγ1-Y783 phosphorylation was determined in 200-μM PKHB1-treated MEC-1 cells using flow cytometry. (B) IP_1_ was quantified in MEC-1 cells left untreated or treated with PKHB1 (200 μM, 2 h). The histogram depicts mean ± SD (*n* = 5). (C) Cell death was analyzed by Annexin-V-positive/PI-positive staining in untreated (control) and PKHB1-treated MEC-1 cells (200 μM, 2 h) pre-incubated with vehicle (−) or the PLC inhibitor U73122. The data are presented as mean ± SD (*n* = 5). (D) The effects of the down-regulation of PLCγ1 on PKHB1-mediated PCD (200 μM, 2 h) were determined in MEC-1 cells transduced with scrambled shRNA (Scr) or two shRNAs against *PLCG1* (shRNA A and B). Cell death, measured by Annexin-V-positive/PI-positive staining, was plotted as mean ± SD (*n* = 5). (E) A representative Ca^2+^ mobilization is illustrated for 200-μM PKHB1-treated cells transduced as described in (D). Ionomycin (1 μM) was used as a control to demonstrate the maximum response. The data are presented as mean ± SD (scrambled shRNA, *n* = 29 cells; shRNA A, *n* = 38 cells; shRNA B, *n* = 33 cells). The statistical analysis included in this figure was performed with the *t*-test.
(TIF)

S9 Fig PKHB1 shows no toxic effect in NSG or C57BL/6 mice. (A) Histological analysis of kidney and liver from NSG mice assessed after the third weekly intraperitoneal injection of vehicle (control) or PKHB1 (10 mg/kg). Two representative liver and kidney images are shown (#1 and #2). Liver sections were stained with hematoxylin-eosin, and kidney sections with periodic acid—Schiff. (B) During 3 wk, C57BL/6 mice received a weekly intraperitoneal injection of vehicle (control) or PKHB1 (10 mg/kg). Mice blood was examined before (−) and 24 h post-injection in a MS9–5 hematological analyzer, and percentages of lymphocytes, monocytes, and neutrophils, as well as the levels of red blood cells, platelets, and hemoglobin, were assessed. Data obtained from vehicle- (white bars) and PKHB1-treated (black bars) mice are plotted as mean ± SD (*n* = 6). (C) Histological analysis of kidney and liver from C57BL/6 mice after the third weekly intraperitoneal injection of vehicle (control) or PKHB1 (10 mg/kg). Two representative liver and kidney images are shown (#1 and #2). Liver and kidney sections were stained as in (A). Compared to vehicle, no sign of toxicity or tissue necrosis was observed in PHKB1-treated NSG or C57BL/6 mice.
(TIF)

## Abbreviations

2-APB: 2-aminoethoxydiphenyl borate
ΔΨ_m_: mitochondrial transmembrane potential
BCR: B cell receptor
CLL: chronic lymphocytic leukemia
ER: endoplasmic reticulum
FISH: fluorescence in situ hybridization
HPLC: high-performance liquid chromatography
IP_3_R: inositol-1, 4, 5-triphosphate receptor
MFI: mean fluorescence intensity
NSG: NOD/scid gamma
PCD: programmed cell death
PI: propidium iodide
QVD: Q-VD-OPh
RT-PCR: real-time PCR
SD: standard deviation
serpase: chymotrypsin-like serine protease
shRNA: short hairpin RNA
TMRE: tetramethylrhodamine ethyl ester
