# Supplementary figures and images for: CD47 Agonist Peptides Induce Programmed Cell Death in Refractory Chronic Lymphocytic Leukemia B Cells via PLCγ1 Activation: Evidence from Mice and Humans
Source: PLoS Med. 2015 Mar 3;12(3):e1001796. doi: 10.1371/journal.pmed.1001796 (PMC4348493; doi:10.1371/journal.pmed.1001796)

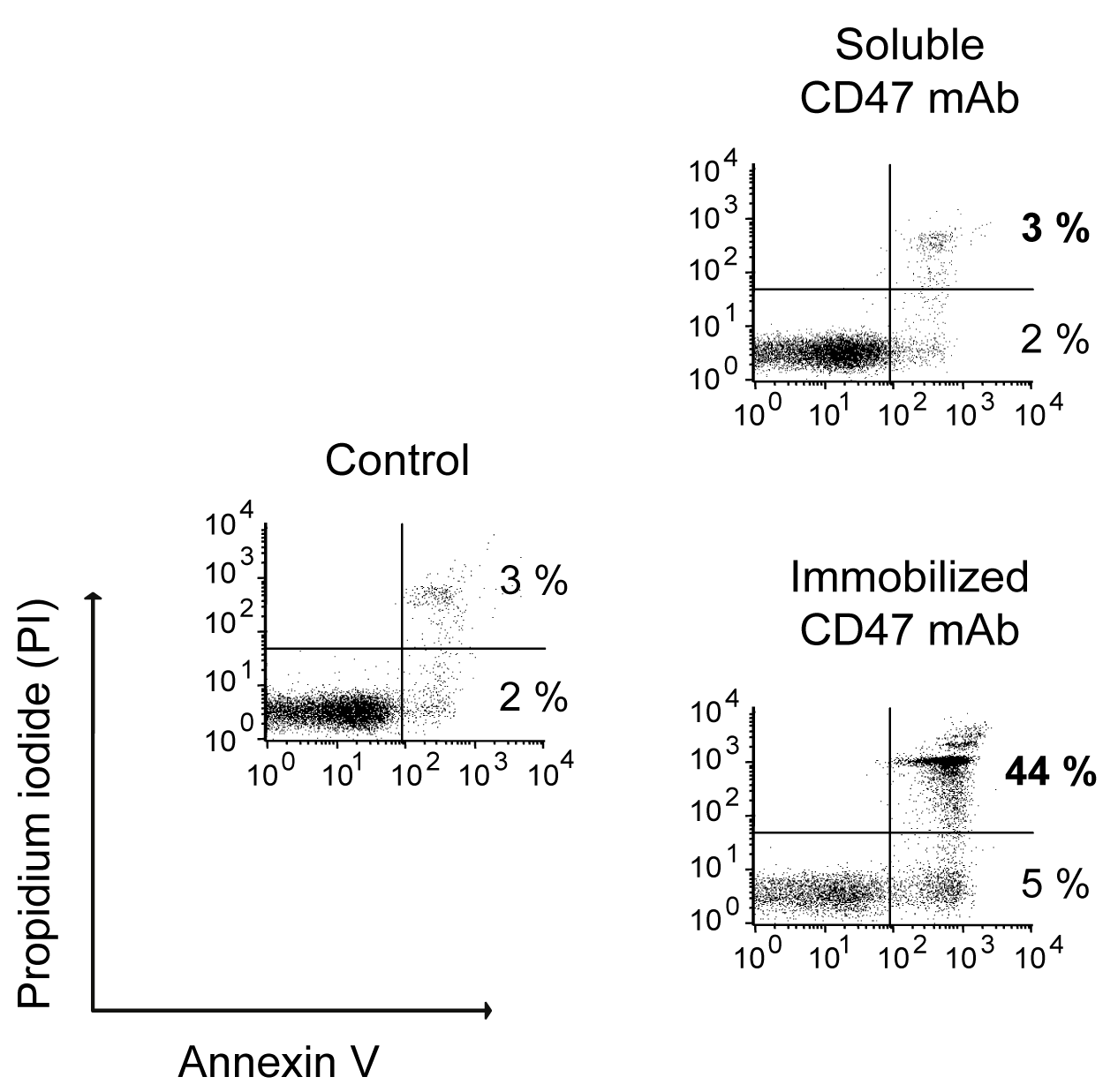

Supplement: S1 Fig — Cell viability measured in CLL B lymphocytes left untreated or incubated for 2 h with CD47 mAb (5 μg/ml, clone B6H12) in soluble or immobilized conditions. The percentages refer to Annexin-V-positive or Annexin-V-positive/PI-positive staining. The cytofluorometric data from a representative CLL patient are shown. This experiment has been done ten times, yielding low interexperimental variability (<5%). (TIF) [file pmed.1001796.s001.tif]

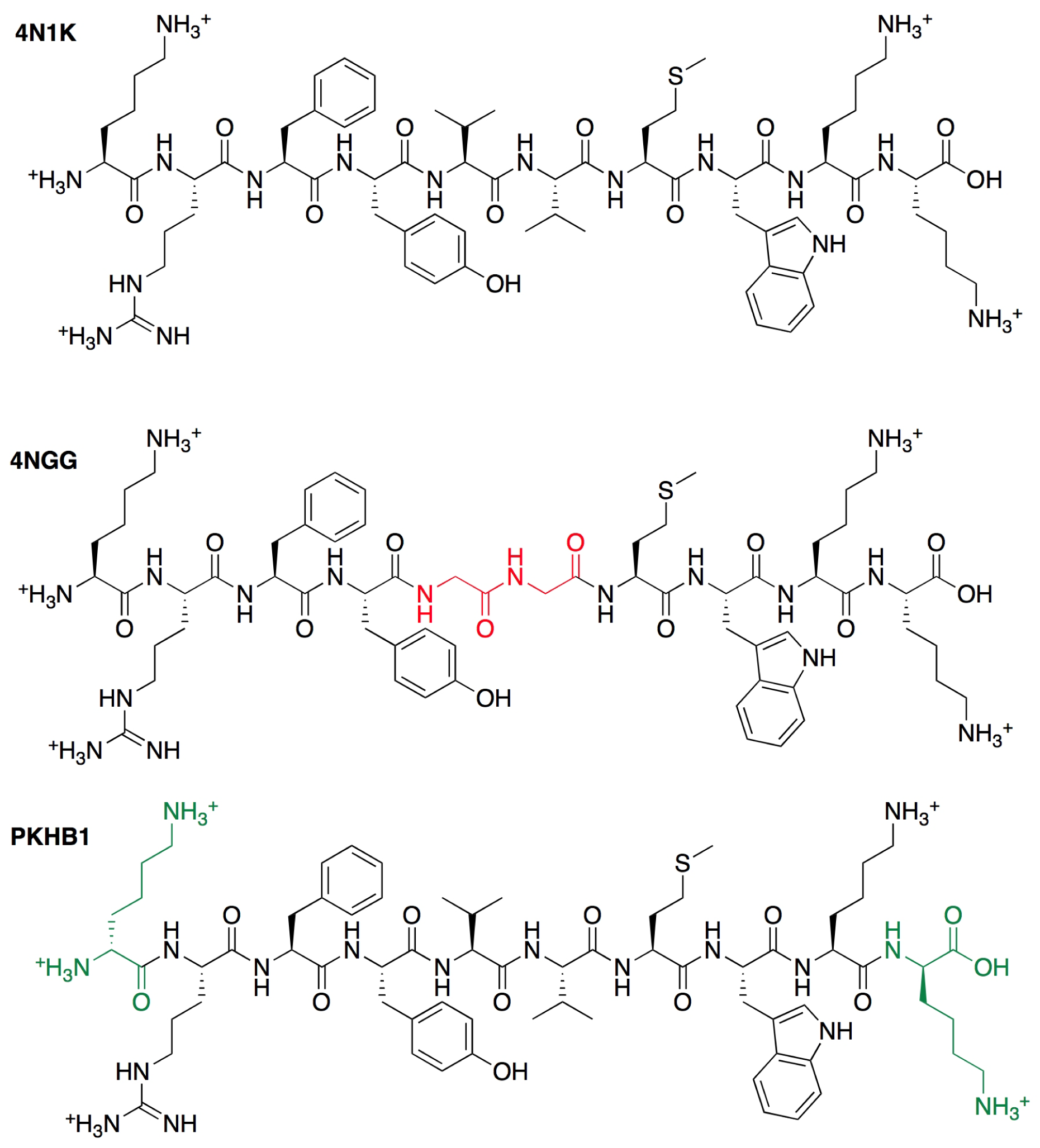

Supplement: S2 Fig — In 4NGG, the two central valines of 4N1K were replaced by glycines (highlighted in red). In PKHB1, the N- and C-terminal lysines of 4N1K were replaced with their D counterparts (highlighted in green). The dissociation constants (K d) of 4NGG, 4N1K, and PKHB1 to CD47—determined by fluorescence polarization, isothermal titration calorimetry, and microscale thermophoresis binding assays [82]—are 1,000,000 nM, 1,500 nM, and 400 nM, respectively. (TIF) [file pmed.1001796.s002.tif]

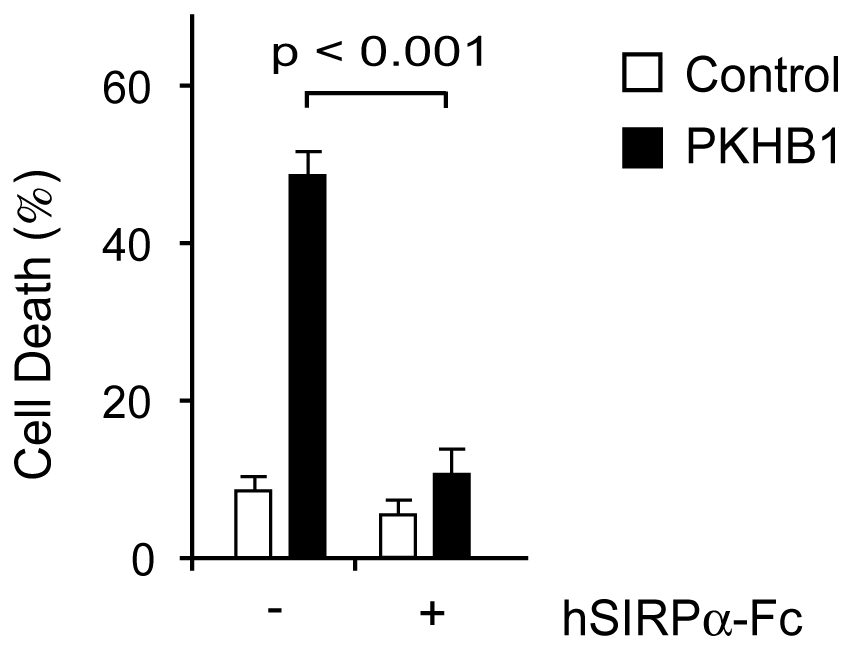

Supplement: S3 Fig — Cell viability was determined in CLL cells that were pre-incubated with vehicle (−) or hSIRPα-Fc (+) and were treated with PKHB1 (200 μM, 2 h). The percentages refer to Annexin-V-positive/PI-positive. The data are graphed as mean ± SD (n = 5). The statistical analysis included in this figure was performed with the t- test. (TIF) [file pmed.1001796.s003.tif]

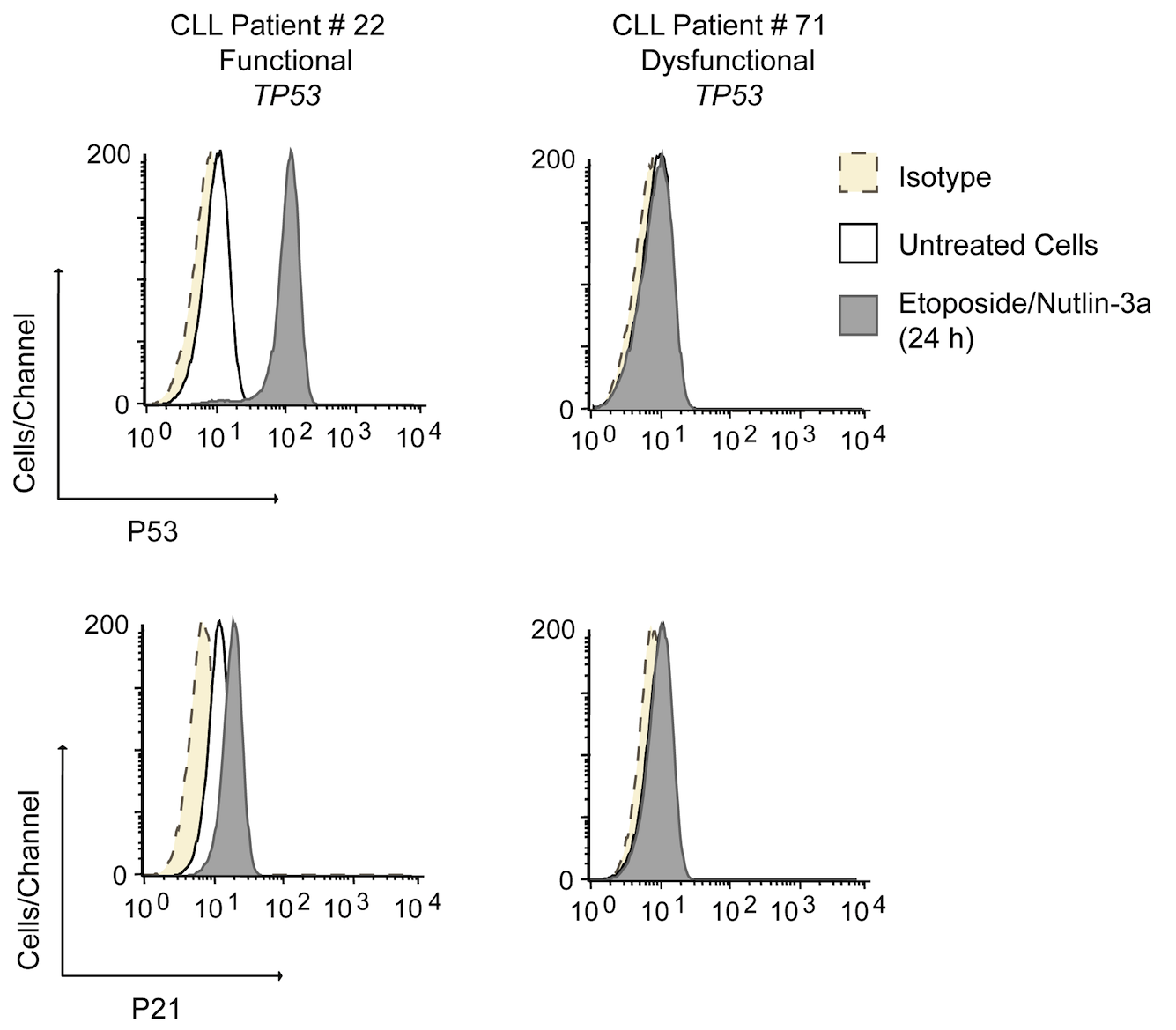

Supplement: S4 Fig — The functional status of TP53 was determined based on the induction of P53 and P21 protein expression by etoposide in combination with nutlin-3a [39]. The upper panels provide representative cytofluorometric plots of P53 in CLL cells from patient #22 (functional TP53, Table 1) and patient #71 (dysfunctional TP53, 96% deletion of 17p; Tables 2 and 3). In a similar experiment, the lower panels depict representative plots of P21 expression in B cells from the same individuals as above. (TIF) [file pmed.1001796.s004.tif]

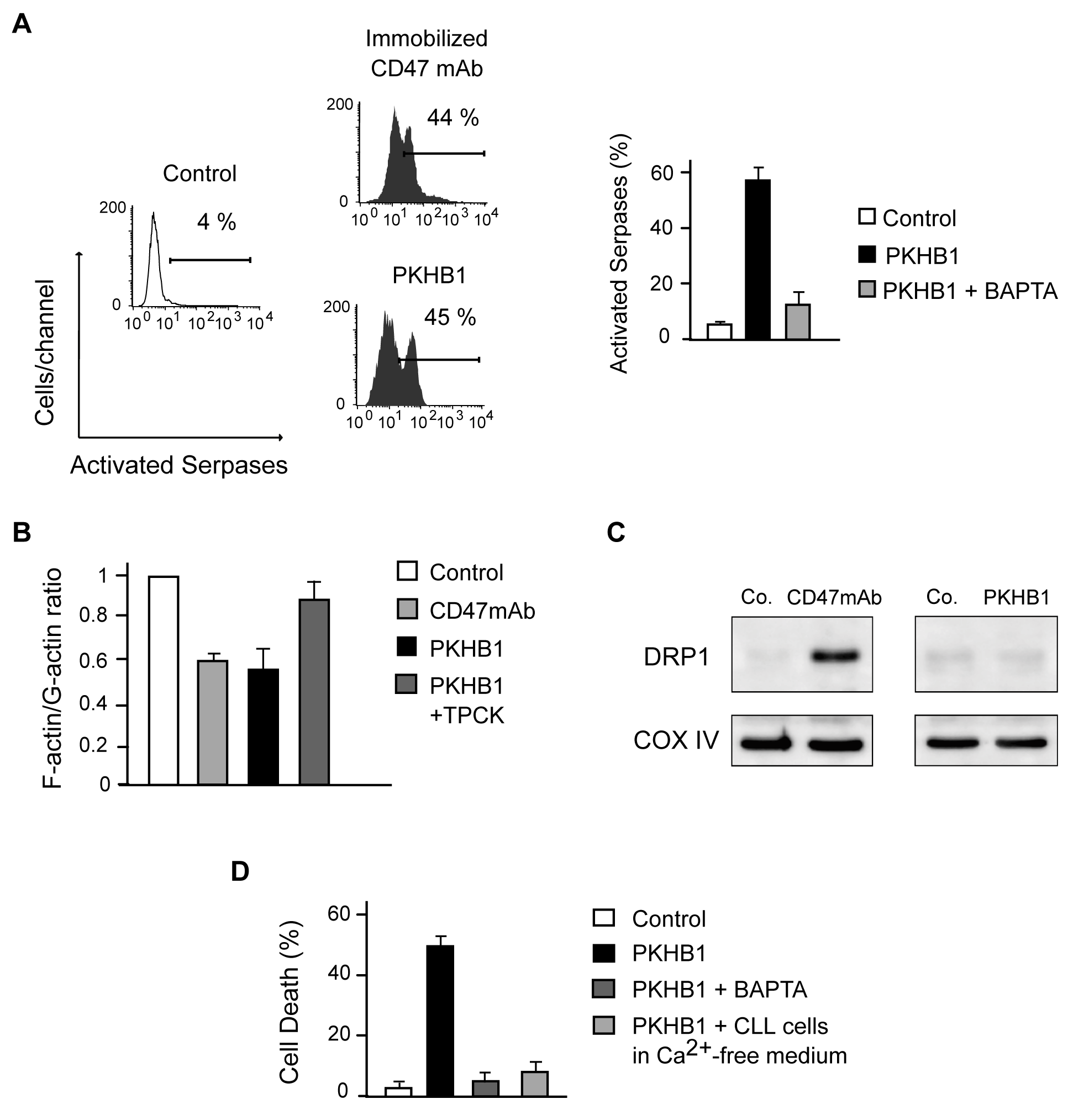

Supplement: S5 Fig — (A) Left: B lymphocytes from a representative CLL donor were left untreated (control) or were incubated for 2 h with immobilized CD47 mAb (B6H12) or PKHB1 (200 μM) before assessment of serpase activity with a green fluorescent SerPase kit (FFCK). The percentages refer to positive staining. Right: B cells from CLL patients (n = 5) were left untreated (control), were incubated with PKHB1 (200 μM, 2 h), or were pre-incubated with the external Ca2+ chelator BAPTA prior to PKHB1 treatment. The data are presented as mean ± SD. (B) CLL cells were exposed to vehicle (control), immobilized CD47 mAb (B6H12, 2 h), or PKHB1 (200 μM, 2 h), and the fluorescence F-actin/G-actin ratio was quantified. One unit refers to the basal F-actin/G-actin ratio scored in control cells. Data are the mean of five independent experiments ± SD. (C) B lymphocytes from a representative CLL donor were left untreated (control) or were incubated for 2 h with either immobilized CD47 mAb (B6H12) or PKHB1 (200 μM) before immunoblot detection of DRP1 in the mitochondrial fraction. Equal loading was confirmed by Cox IV probing. (D) Cell death was measured by Annexin-V-positive/PI-positive staining in PKHB1-treated CLL B cells (200 μM, 2 h) pre-incubated with vehicle, the external Ca2+ chelator BAPTA, or Ca2+-free medium (n = 5). The data are graphed as mean ± SD. (TIF) [file pmed.1001796.s005.tif]

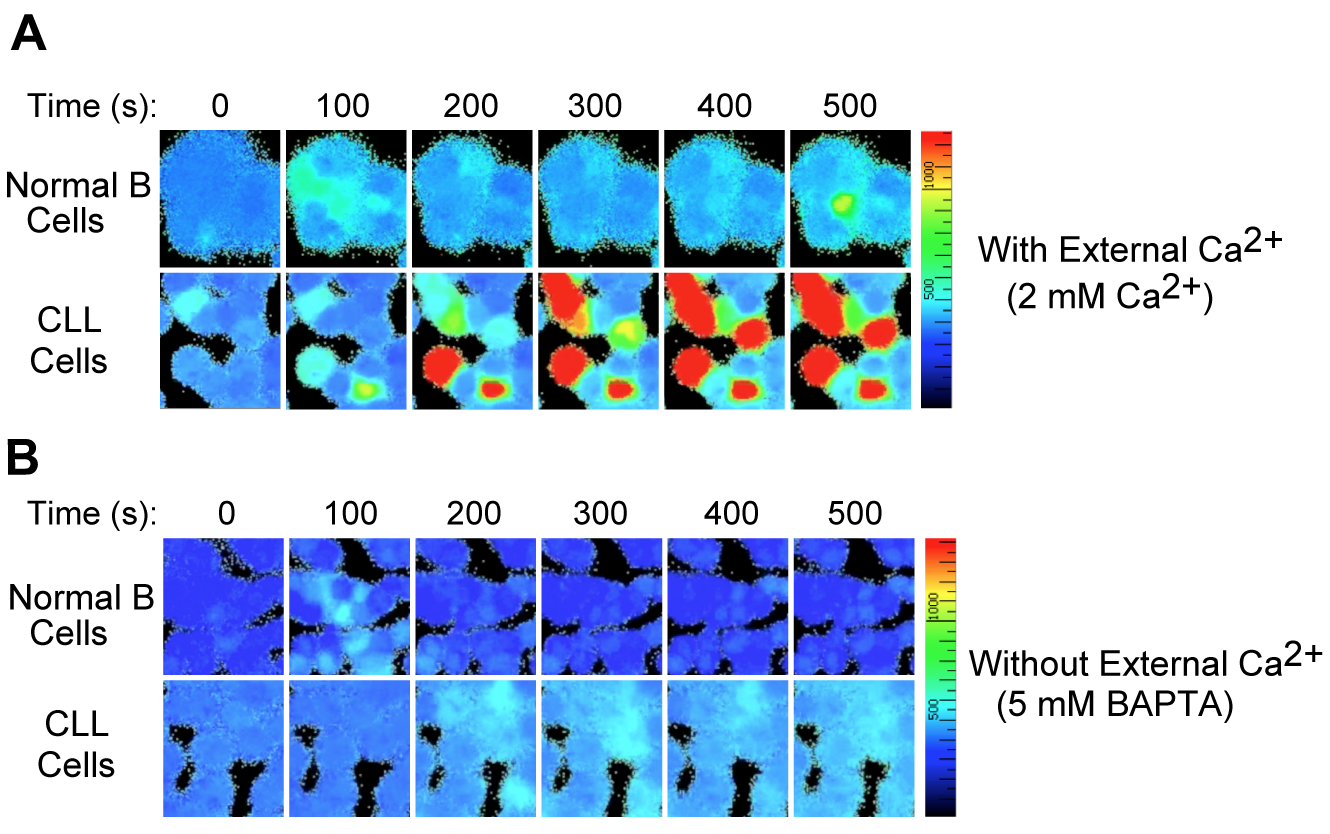

Supplement: S6 Fig — Normal B cells from a healthy donor and B lymphocytes from a representative CLL patient were stained with Fura2-AM and pluronic acid in glass bottom dishes. The cells were treated with PKHB1 (200 μM) and imaged using a dual excitation fluorometric imaging system for the indicated time. Cytosolic Ca2+ variations were recorded in the presence of 2 mM Ca2+ (A) or 5 mM BAPTA (B). The scale bar depicts the relative Ca2+ intensity. (TIF) [file pmed.1001796.s006.tif]

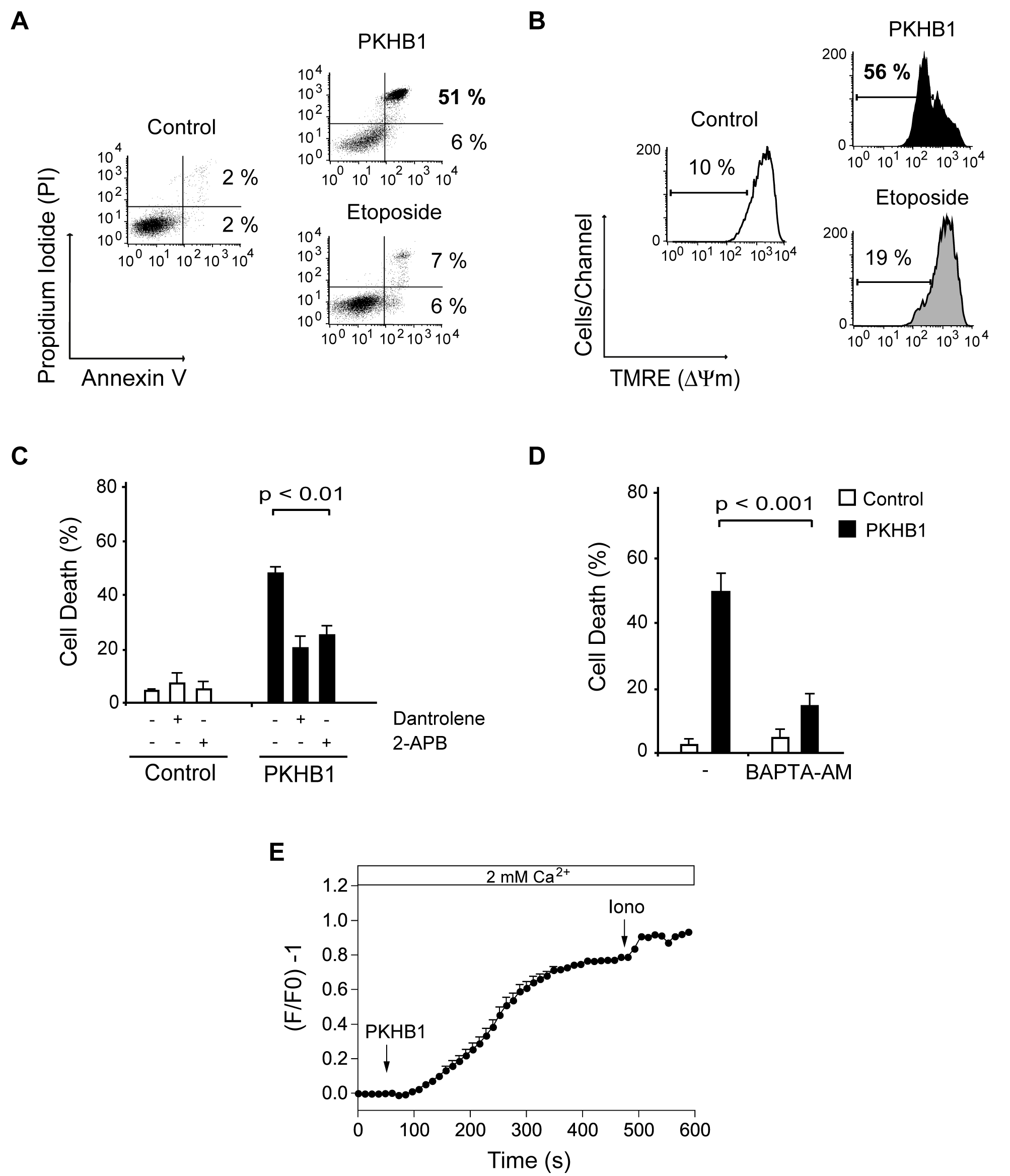

Supplement: S7 Fig — (A) MEC-1 cells were left untreated or were incubated for 2 h with PKHB1 (200 μM) or for 12 h with the P53-dependent cell death inducer etoposide (250 μM); the cells were then labeled with Annexin-V and PI to assess cell viability. The percentages refer to Annexin-V-positive or Annexin-V-postsitive/PI-positive staining. (B) MEC-1 cells were incubated as in (A) and stained with TMRE, and ΔΨm was determined by flow cytometry. The percentages refer to cells with ΔΨm loss. As depicted in (A) and (B), the dysfunctional TP53 MEC-1 cell line was resistant to etoposide (12 h of treatment). (C) Cell death was measured by Annexin-V-positive/PI-positive labeling in untreated (control) or PKHB1-treated MEC-1 cells (200 μM, 2 h) pre-incubated with vehicle (−) or the ER receptor inhibitors dantrolene or 2-APB. The data are presented as mean ± SD (n = 6). (D) As described in (C), cell death was measured in untreated (control) or 200-μM PKHB1-treated MEC-1 cells pre-incubated with vehicle (−) or the intracellular Ca2+ chelator BAPTA-AM. The data, which refer to Annexin-V and PI co-positivity, are mean ± SD (n = 5). (E) A representative Ca2+ mobilization triggered by 200 μM PKHB1 in MEC-1 cells is illustrated. Ionomycin (Iono, 1 μM) was used as a control to demonstrate the maximum response. The data are presented as mean ± SD (n = 26 cells). The statistical analysis included in this figure was performed with the t-test. (TIF) [file pmed.1001796.s007.tif]

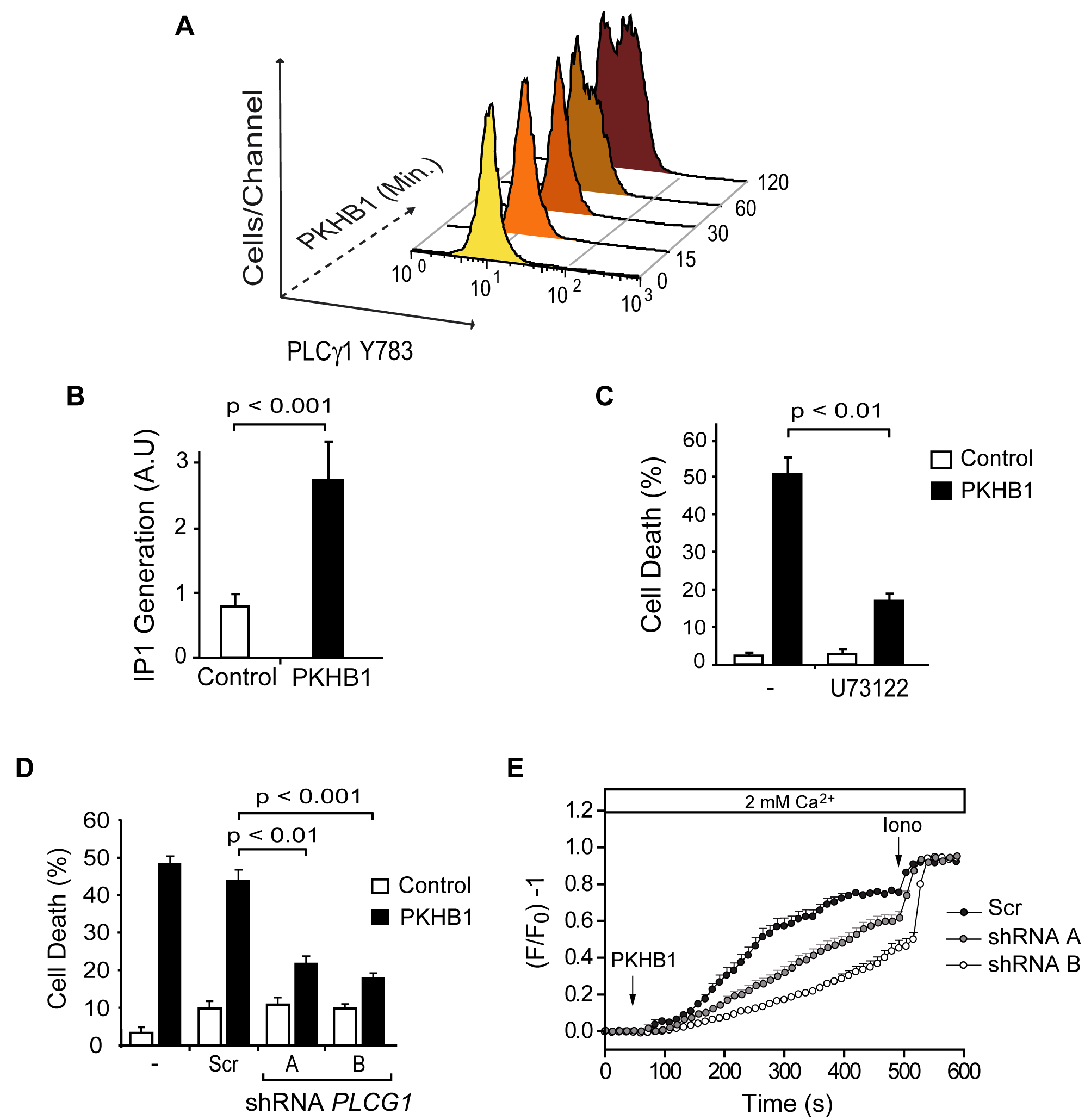

Supplement: S8 Fig — (A) At the indicated time, PLCγ1-Y783 phosphorylation was determined in 200-μM PKHB1-treated MEC-1 cells using flow cytometry. (B) IP1 was quantified in MEC-1 cells left untreated or treated with PKHB1 (200 μM, 2 h). The histogram depicts mean ± SD (n = 5). (C) Cell death was analyzed by Annexin-V-positive/PI-positive staining in untreated (control) and PKHB1-treated MEC-1 cells (200 μM, 2 h) pre-incubated with vehicle (−) or the PLC inhibitor U73122. The data are presented as mean ± SD (n = 5). (D) The effects of the down-regulation of PLCγ1 on PKHB1-mediated PCD (200 μM, 2 h) were determined in MEC-1 cells transduced with scrambled shRNA (Scr) or two shRNAs against PLCG1 (shRNA A and B). Cell death, measured by Annexin-V-positive/PI-positive staining, was plotted as mean ± SD (n = 5). (E) A representative Ca2+ mobilization is illustrated for 200-μM PKHB1-treated cells transduced as described in (D). Ionomycin (1 μM) was used as a control to demonstrate the maximum response. The data are presented as mean ± SD (scrambled shRNA, n = 29 cells; shRNA A, n = 38 cells; shRNA B, n = 33 cells). The statistical analysis included in this figure was performed with the t-test. (TIF) [file pmed.1001796.s008.tif]

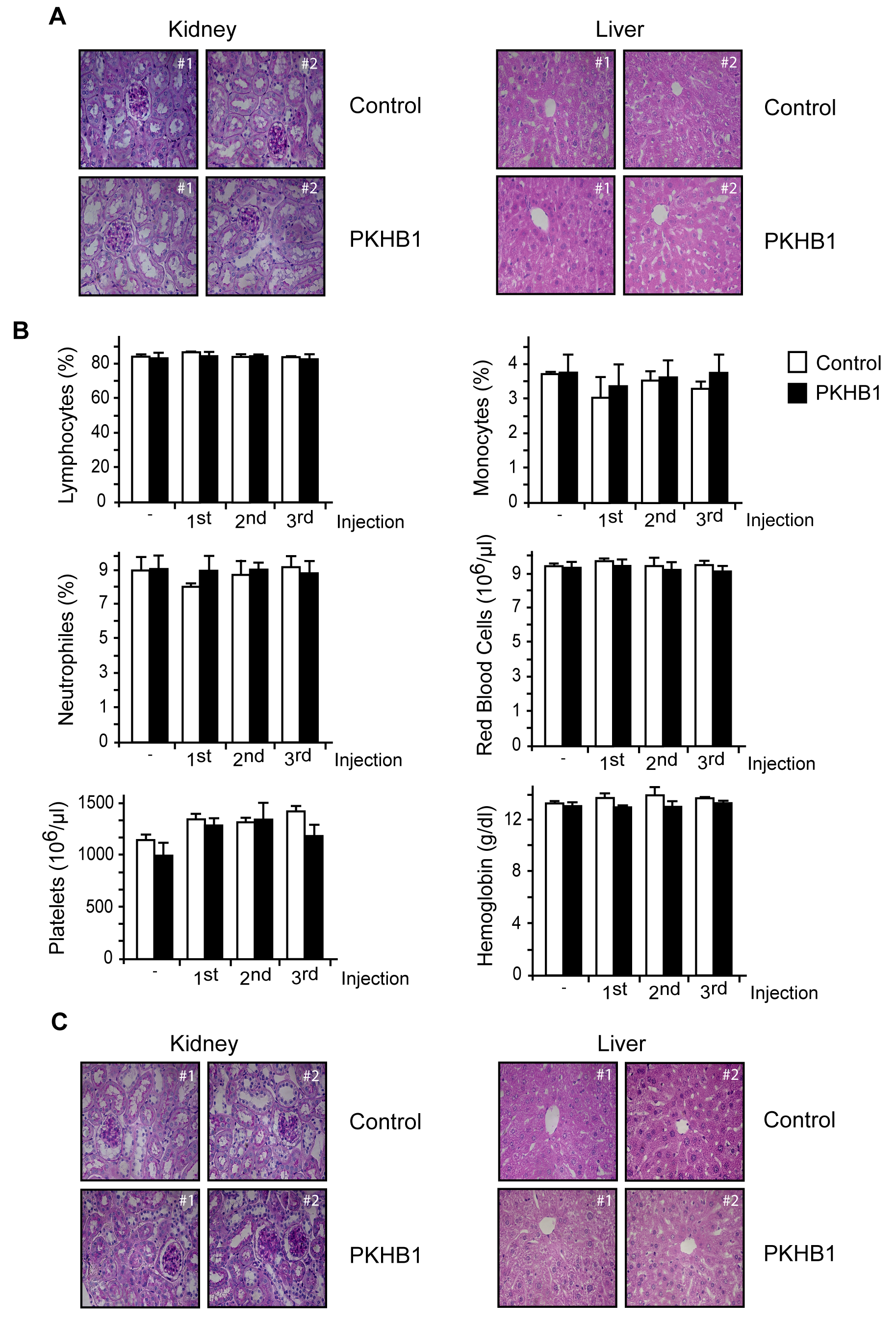

Supplement: S9 Fig — (A) Histological analysis of kidney and liver from NSG mice assessed after the third weekly intraperitoneal injection of vehicle (control) or PKHB1 (10 mg/kg). Two representative liver and kidney images are shown (#1 and #2). Liver sections were stained with hematoxylin-eosin, and kidney sections with periodic acid—Schiff. (B) During 3 wk, C57BL/6 mice received a weekly intraperitoneal injection of vehicle (control) or PKHB1 (10 mg/kg). Mice blood was examined before (−) and 24 h post-injection in a MS9–5 hematological analyzer, and percentages of lymphocytes, monocytes, and neutrophils, as well as the levels of red blood cells, platelets, and hemoglobin, were assessed. Data obtained from vehicle- (white bars) and PKHB1-treated (black bars) mice are plotted as mean ± SD (n = 6). (C) Histological analysis of kidney and liver from C57BL/6 mice after the third weekly intraperitoneal injection of vehicle (control) or PKHB1 (10 mg/kg). Two representative liver and kidney images are shown (#1 and #2). Liver and kidney sections were stained as in (A). Compared to vehicle, no sign of toxicity or tissue necrosis was observed in PHKB1-treated NSG or C57BL/6 mice. (TIF) [file pmed.1001796.s009.tif]
